# Supplementary material for: Gene-repressing epigenetic reader EED unexpectedly enhances cyclinD1 gene activation
Source: Mol Ther Nucleic Acids. 2023 Feb 21;31:717–29. doi: 10.1016/j.omtn.2023.02.024 (PMC10009644; doi:10.1016/j.omtn.2023.02.024)
Supplement: Document S1. Figures S1–S8 and Tables S1–S5 [file mmc1.pdf]

OMTN, Volume *31*

Supplemental information

Gene-repressing epigenetic reader EED unexpectedly enhances cyclinD1 gene activation

Mengxue Zhang, Jing Li, Qingwei Wang, Go Urabe, Runze Tang, Yitao Huang, Jose Verdezoto Mosquera, K. Craig Kent, Bowen Wang, Clint L. Miller, and Lian-Wang Guo

Supplemental Figures

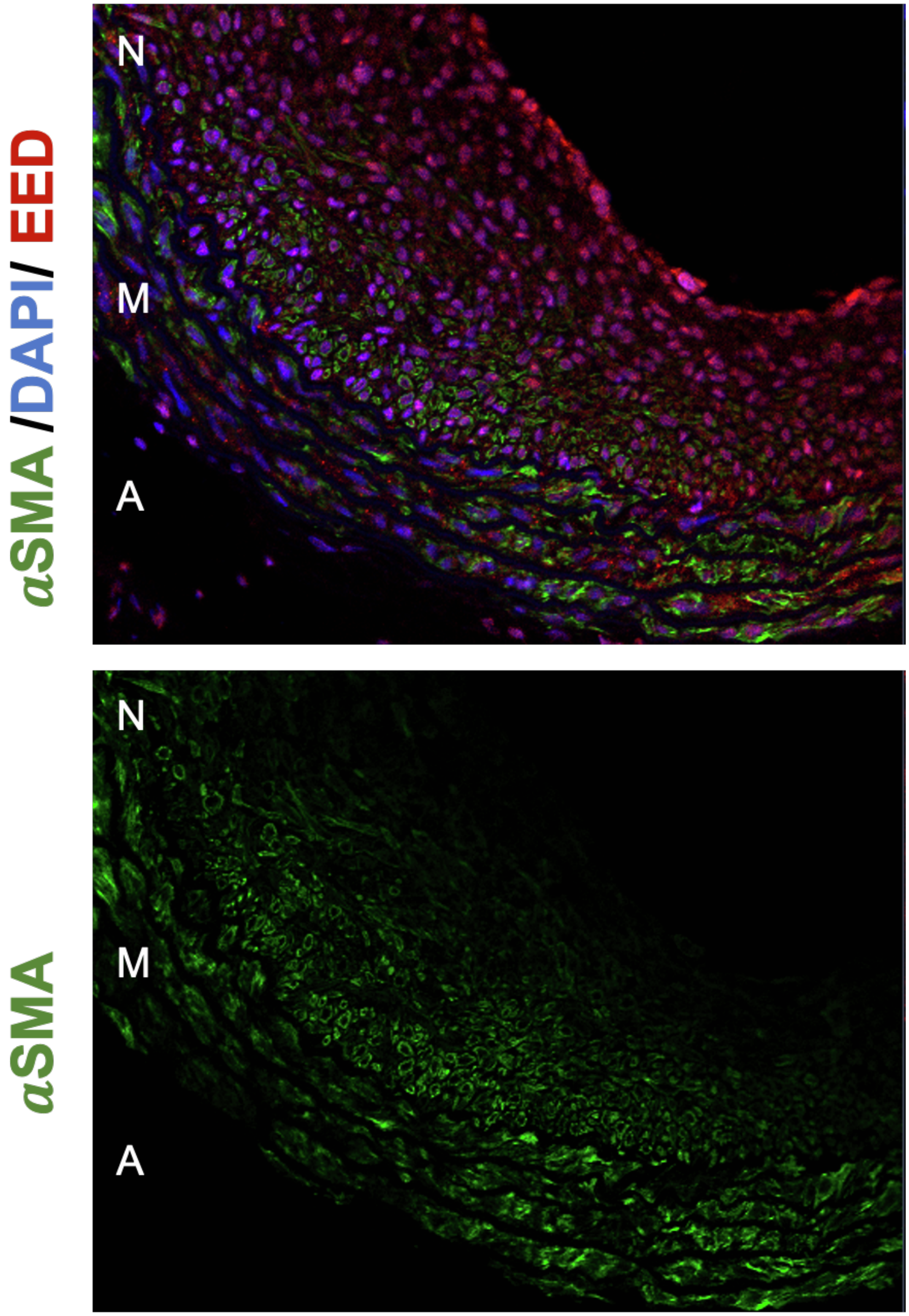

Figure S1. Enlarged view of EED and αSMA co-staining.
Please refer to the Figure 1C confocal images. The picture of merged colors shows EED in the nucleus surrounded by αSMA in the cytosol.

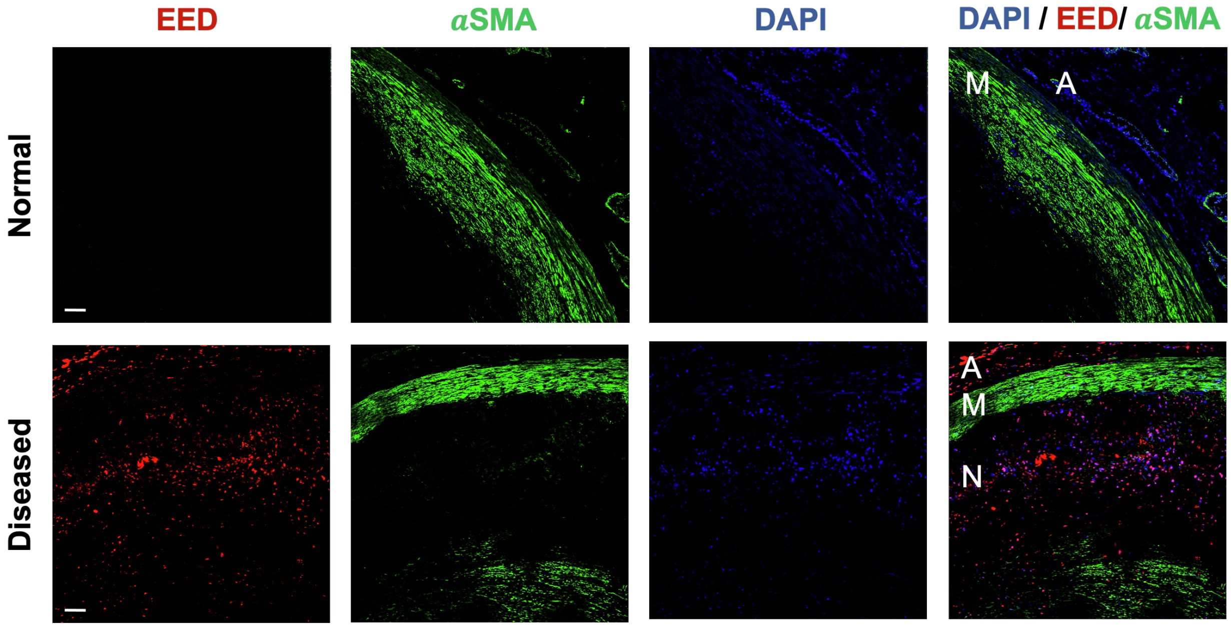

Figure S2. Co-staining of EED and αSMA.
Please refer to the Figure 2D confocal images. Shown here are all the images of individual color channels.

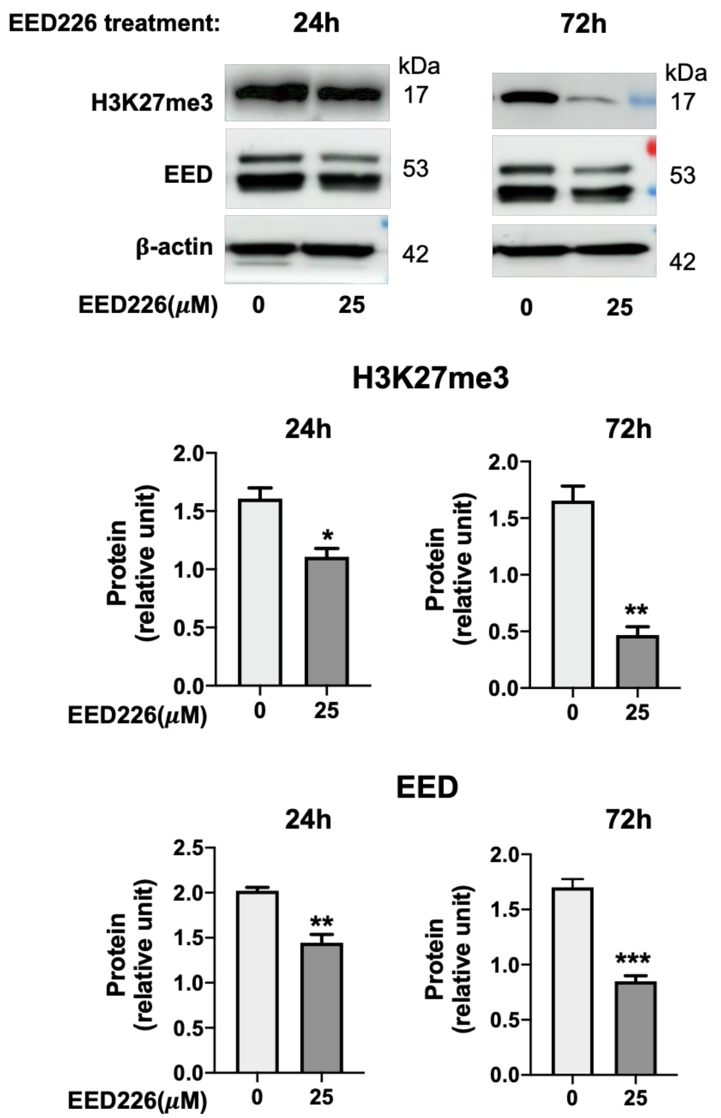

Figure S3. EED-selective inhibitor EED226 reduces SMC H3K27me3 levels
Starved MOVAS mouse SMCs were incubated with EED226 (25 μM) or equal amount of DMSO (0 μM) for indicated hours before harvest for immunoblot analysis. Student's t-test: *P<0.05, **P<0.01, ***P<0.001.

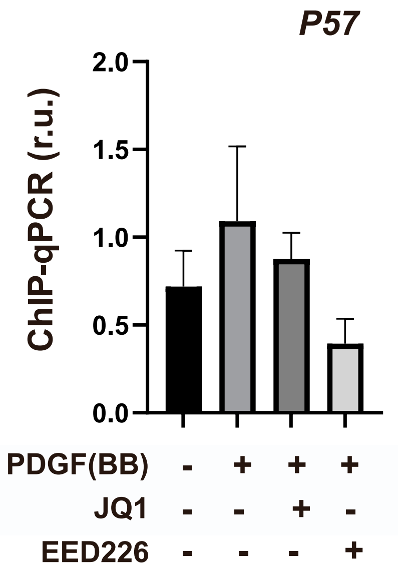

Figure S4. ChIP-qPCR to detect the distal side of the *P57* promoter

MOVAS cells were cultured, starved, preincubated with EED226 or JQ1 (1 μM), and then stimulated with PDGF-BB. Cells were harvested for assays after 48h PDGF stimulation. ChIP was performed using an antibody specific for endogenous EED or endogenous BRD4, as described for Figure 6. qPCR was performed using primers for a region on the distal side of the *P57* promoter (~ -2034bp-1885bp from TSS). Quantification: Three independent ChIP experiments were performed in separate times (n = 3). The qPCR reading was first normalized to *Gapdh* using the delta-delta ct method, and then to the basal-condition (no PDGF-BB, no inhibitor) value from one of the 3 repeat experiments. All readings were thereby converted to fold changes and averaged to produce mean ± SEM. Statistics: One-way ANOVA and Bonferroni post-hoc test. No significance was found ($P > 0.05$).

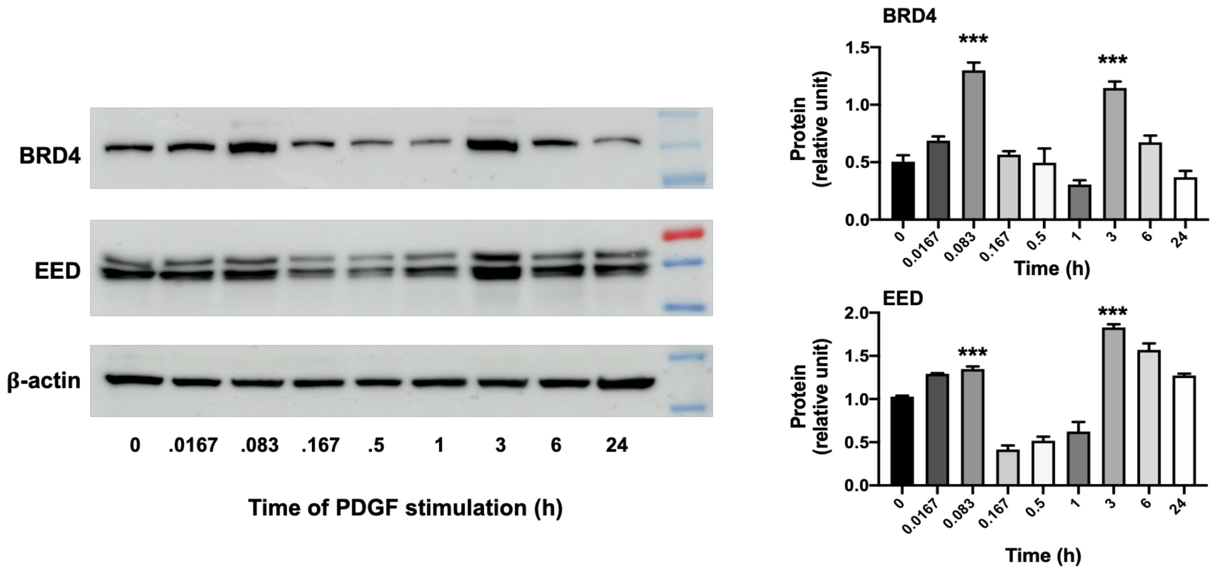

Figure S5. PDGF-induced EED and BRD4 protein level changes in SMCs
Starved MOVAS mouse SMCs were stimulated with 20 ng/ml PDGF-BB for indicated hours before immunoblotting assay. Densitometry was normalized to loading control (β-actin). The data values from 3 independent repeat experiments were averaged to calculate mean ± SEM (n = 3). Statistics: One-way analysis of variance (ANOVA) followed by Bonferroni post-hoc test; ***$P<0.001$, compared to the basal condition without PDGF-BB (the first bar in each plot).

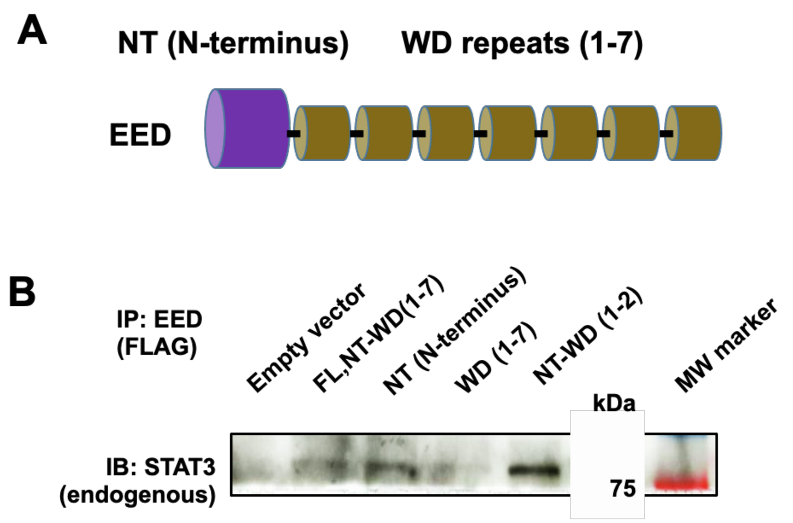

Figure S6. The **EED N-terminus is mainly responsible for its interaction with STAT3**

A. Diagram of the domains of the EED protein.
B. B. Co-IP. FLAG-tagged EED constructs were expressed in HEK293A cells, and an anti-FLAG antibody was used for IP. An antibody for endogenous STAT3 was used for immunoblotting (IB). FL, full length, including the N-terminus (NT) and 7 WD repeats.

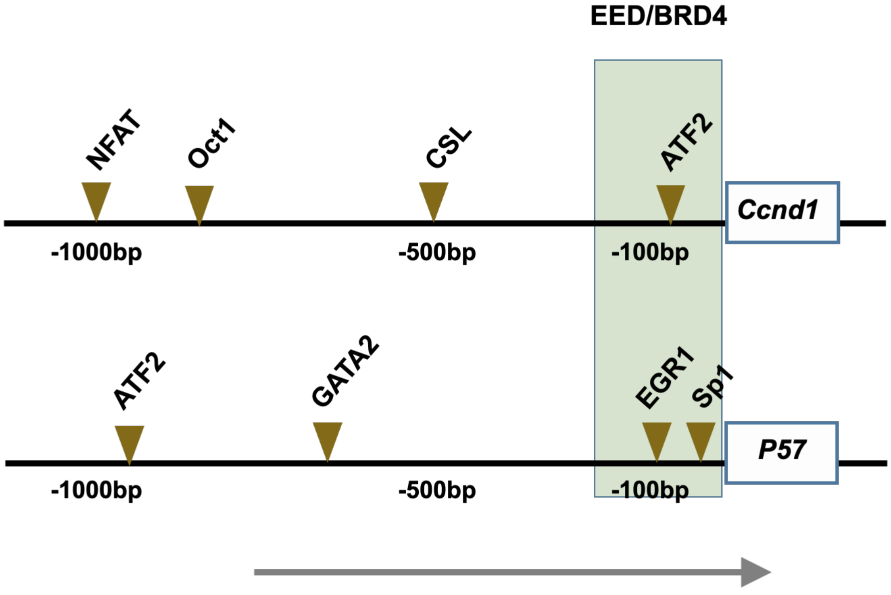

Figure S7. Transcription factors that possibly bind *Ccnd1* and *P57* promoters

The box marks the promoter regions where EED and BRD4 are enriched.

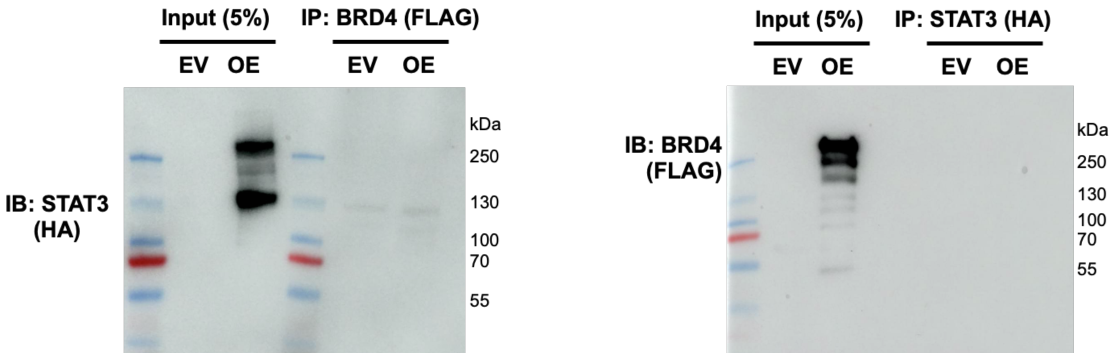

Figure S8. Lack of BRD4/EZH2 co-immunoprecipitation

The assay was performed as described for Figure 3. Briefly, HEK293 cells were transfected with plasmids detailed in Methods. An anti-HA or anti-FLAG antibody was used for IP or the corresponding IB. EV: empty vector.

Supplemental Tables

Table S1. Major resources

Product	Manufacturer	Catalog Number
MOVAS cells	ATCC	CRL-2797
DMEM	Thermo Fisher Scientific	10313039
PDGF-BB	R&D	520-BB
Lipofectamine RNAiMAX Transfection Reagent	Invitrogen	3778150
JetPRIME Transfection reagent	Polyplus transfection	114-07
PageRuler™ PLUS Prestained Protein Ladder	Thermo Fisher Scientific	26620
Clarity Western ECL Substrate	Bio-Rad	170-5060
QIAGEN Plasmid MIDIprep Kit	QIAGEN	12143
PureLink™ Quick Plasmid Miniprep Kit	Invitrogen	K210011
PureLink™ Quick Gel Extraction and PCR Purification Combo Kit	Invitrogen	K220001
lentiCRISPR v2	addgene	52961
Lenti-x qRT-PCR Titration Kit	Takara	631235
Trizol Reagent	Thermo Fisher Scientific	15596026
Perfecta SYBR Green Master Fast Mix	VWR	101414-286

Pierce™ Protein A/G Magnetic Agarose Beads	Invitrogen	78610
UltraPure™ Salmon Sperm DNA Solution	Invitrogen	15632011
CellTiter-Glo kit	Promega	G7570
Anti-HA Magnetic Agarose beads	Invitrogen	88836
Pierce™ Anti-DYKDDDDK (FLAG) Magnetic Agarose	Thermo Fisher Scientific	A36797
Mouse IgG	Invitrogen	10400C
High-Capacity cDNA Reverse Transcription kit	Thermo Fisher Scientific	4368814
Pierce BCA Protein Assay kit,	Thermo Fisher Scientific,	23227
EED226 (in vitro use)	Selleckchem	S8496
EED226 (in vivo use)	Chemietek	CT-EED226
A-395	Sigma-Aldrich	SML1923
JQ1	Apexbio	A1910

Table S2. sgRNAs and siRNAs for knockdown

Name	Sequence (5’-3’)
EED sgRNA	Sense: GGAAGTGTCGACTGCGCCGG
	Antisense: CCGGCGCAGTCGACACTTCC
STAT3 shRNA#1	Target Sequence: CCAACATCTGCCTGGACCGTCTG
STAT3 shRNA#2	Target Sequence: TCAGGTTGCTGGTCAAATTTC
STAT3 shRNA#3	Target Sequence: ACATGGAGGAGTCTAACAA

Table S3. Antibodies for Western blot (WB), ChIP, IP, or immunofluorescence (IF)

Antigen	Manufacturer	Catalog Number	Dilution Ratio	Application
Beta-actin	Proteintech	60008-1-Ig	1:10000	WB
FLAG tag	Sigma-Aldrich	F3165	1:1000	WB/IP
HA tag	Cell Signaling Technology	3724	1:1000	WB/IP
Histone-3	Cell Signaling Technology	14269	1:1000	WB
H3K27me3	Cell Signaling Technology	4909	1:1000	WB
H3K27ac	Active Motif	39133	1:1000	WB
BRD4	Abcam	Ab128874	1:1000	WB
BRD4	Abcam	Ab128874	1:1000	ChIP
EED	R&D	AF5827	1:500	WB
EED	Cell Signaling Technology	85322S	1:1000	ChIP
EED	LSBio	LS-C153641	1:100	IF
EZH2	Cell Signaling Technology	5246	1:1000	WB
STAT3	Cell Signaling Technology	9139	1:1000	WB
P-STAT3	Cell Signaling Technology	9145	1:1000	WB
Cyclin-D1	Cell Signaling Technology	55506	1:1000	WB
Cyclin-D1	Cell Signaling Technology	55506	1:100	IF
p-STAT3	Novus	NB100-82213	1:100	IF

Table S4. Primers for ChIP-qPCR

Mouse *Ccnd1*<br>(Region: -176 to -18 bp)	Forward: GGGGAGTTTTGTTGAAGTTGC
	Reverse: GGGCTGTGGTCTCGGTTG
Mouse *P57*<br>(Region: ~ -183 to -2 bp)	Forward: AGTGCGCTGTGCTCGAGG
	Reverse: GTCGAAGGCTGTGCAAACG
Mouse *P57*<br>(Region: ~ -2034 to -1885 bp)	Forward: TGCTTCTCCCAACAAATAG
	Reverse: CTCCCCAACAGTCCAAAT
Mouse *Gapdh*	Forward: AGGTCGGTGTGAACGGATTTG
	Reverse: TGTAGACCATGTAGTTGAGGTCA

Table S5. Primers for qRT-PCR

Ccnd1(CyclinD1)	Forward: AGAGGCGGATGAGAACAAGC
	Reverse: CCTTGTTTAGCCAGAGGCCG
Cdkn1c (P57)	Forward: GCCAATGCGAACGACTTCTT
	Reverse: GTTTGGAGAGGGACACCCTG
Gapdh	Forward: GAGAGTGTTTCCTCGTCCCG
	Reverse: ATGGGCTTCCCGTTGATGAC
Stat3	Forward: GGGCCTGGTGTGAACTACTC
	Reverse: ACATCGGCAGGTCAATGGTA
