# Supplementary material for: Gene-repressing epigenetic reader EED unexpectedly enhances cyclinD1 gene activation
Source: Mol Ther Nucleic Acids. 2023 Feb 21;31:717–29. doi: 10.1016/j.omtn.2023.02.024 (PMC10009644; doi:10.1016/j.omtn.2023.02.024)
Supplement: Document S2. Article plus supplemental information [file mmc2.pdf]

Molecular Therapy
Nucleic Acids
Original Article

Gene-repressing epigenetic reader EED unexpectedly enhances cyclinD1 gene activation

Mengxue Zhang,[1,7] Jing Li,[1,7] Qingwei Wang,[1,7] Go Urabe,[1] Runze Tang,[1] Yitao Huang,[1] Jose Verdezoto Mosquera,[2,3] K. Craig Kent,[1] Bowen Wang,[1] Clint L. Miller,[2,3,4,5] and Lian-Wang Guo[1,5,6]

[1]Department of Surgery, School of Medicine, University of Virginia, Charlottesville, VA 22908, USA; [2]Department of Biochemistry and Molecular Genetics, University of Virginia, Charlottesville, VA 22908, USA; [3]Center for Public Health Genomics, University of Virginia, Charlottesville, VA 22908, USA; [4]Department of Public Health Sciences, University of Virginia, Charlottesville, VA 22908, USA; [5]Robert M. Berne Cardiovascular Research Center, University of Virginia, Charlottesville, VA 22908, USA; [6]Department of Molecular Physiology and Biological Physics, School of Medicine, University of Virginia, Charlottesville, VA 22908, USA

Epigenetically switched, proliferative vascular smooth muscle cells (SMCs) form neointima, engendering stenotic diseases. Histone-3 lysine-27 trimethylation (H3K27me3) and acetylation (H3K27ac) marks are associated with gene repression and activation, respectively. The polycomb protein embryonic ectoderm development (EED) reads H3K27me3 and also enhances its deposition, hence is a canonical gene repressor. However, herein we found an unexpected role for EED in activating the *bona fide* pro-proliferative gene *Ccnd1* (cyclinD1). EED overexpression in SMCs increased *Ccnd1* mRNA, seemingly contradicting its gene-repressing function. However, consistently, EED co-immunoprecipitated with gene-activating H3K27ac reader BRD4, and they co-occupied at both mitogen-activated *Ccnd1* and mitogen-repressed *P57* (*bona fide* anti-proliferative gene), as indicated by chromatin immunoprecipitation qPCR. These results were abolished by an inhibitor of either the EED/H3K27me3 or BRD4/H3K27ac reader function. In accordance, elevating BRD4 increased H3K27me3. *In vivo*, while EED was upregulated in rat and human neointimal lesions, selective EED inhibition abated angioplasty-induced neointima and reduced cyclinD1 in rat carotid arteries. Thus, results uncover a previously unknown role for EED in *Ccnd1* activation, likely via its cooperativity with BRD4 that enhances each other's reader function; i.e., activating pro-proliferative *Ccnd1* while repressing anti-proliferative *P57*. As such, this study confers mechanistic implications for the epigenetic intervention of neointimal pathology.

INTRODUCTION

Smooth muscle cell (SMC), the formative cell type of the vascular wall, is key to maintaining vascular tone in healthy arteries.[1] However, in a pathogenic environment, SMCs transition to a proliferative state contributing to the formation of flow-obstructive neointimal lesions that occupy the lumen, a process termed neointimal hyperplasia (IH).[2] IH is a hallmark of atherosclerotic plaque and the principal cause of recurrent stenosis that spoils treatments of atherosclerosis such as angioplasty/stenting and vein grafting.[2] Given that atherosclerosis and recurrent disease comprise the leading cause of mortality and morbidity globally,[3] it is imperative to better understand the inner workings of SMC/neointima proliferation as a basis for developing improved therapies.

The SMC phenotypic transition to a proliferative state is essentially rendered by chromatin remodeling in response to micro-environmental stimulations; e.g., angioplasty injury and exposure to platelet-derived growth factors (PDGFs). Environmental perturbations imprint bookmarks on the chromatin, including histone-3 lysine 27 acetylation (H3K27ac) and trimethylation (H3K27me3).[4] These marks are added by enzymes dubbed as epigenetic writers such as enhancer of zeste homolog 2 (EZH2) and removed by eraser enzymes. While the epigenetic landscape is dynamically reshaped by writers and erasers, the histone code (a collection of bookmarks) is interpreted by readers to effectuate transcriptional reprogramming.[5] Writer and eraser enzymes have broad substrates including non-histone proteins. In contrast, readers are primarily chromatin-associated.[6] Importantly, they often couple with transcription factors, cofactors, and regulatory DNA and RNA elements in a combinatorial manner to assume functional specificity, thereby precipitating context-dependent (e.g., cell type, stimulant) cell state transitions.[5,7] As such, readers emerge as attractive epigenomic targets for precision therapy, with inhibitors being rapidly developed to treat cancers in human trials.[8,9] However, their mechanistic interplay and contribution thereof to SMC proliferation and IH remain poorly understood.

Received 18 December 2021; accepted 16 February 2023;
https://doi.org/10.1016/j.omtn.2023.02.024.

[7]These authors contributed equally

Correspondence: Clint L. Miller, Ph.D., Center for Public Health Genomics, MSB 3231, PO Box 800717, Charlottesville, VA 22908, USA.
E-mail: clintm@virginia.edu
Correspondence: Lian-Wang Guo, Ph.D. Department of Surgery, School of Medicine, University of Virginia 409 Lane Rd, Charlottesville, VA 22908, USA.
E-mail: lg8zr@virginia.edu

This is an open access article under the CC BY license (http://creativecommons.org/licenses/by/4.0/).

Chemical modifications at H3K27 are key to opened and closed chromatin states corresponding to gene activation and repression, respectively. Although H3K27ac opens local chromatin structure allowing for the access of transcription machinery and gene expression, the H3K27me3 mark imparts an opposite effect.[4] Bromodomain protein 4 (BRD4) is a gene-activating H3K27ac reader and powerful pathophysiological regulator.[10] Our and others' recent work revealed BRD4 as a determinant of vascular SMC/neointima proliferation.[11,12] On the contrary, embryonic ectoderm development (EED), an H3K27me3 reader,[13] is gene-repressive as a core component of the polycomb repressive complex 2 (PRC2).[14] While its importance in oncology has attracted considerable interest and prompted pharmaceutical tests,[15] its role in IH was not known. Moreover, an intriguing question in chromatin biology is whether EED and BRD4, the two seemingly opposing readers, cooperate to fulfill their functions.

Herein we observed that selective EED inhibition reduced IH in an *in vivo* rat model of angioplasty. We further investigated an EED-mediated mechanism in promoting SMC proliferation. Paradoxically, our data showed a positive role of EED in *Ccnd1* (cyclinD1) activation, contradicting the known EED function of gene repression.[14,16] Further analysis revealed that EED and BRD4 formed a complex, which was dependent upon their chromatin-associated reader function. Remarkably, immunoprecipitation (IP) of either EED or BRD4 could pull down DNA fragments from both *Ccnd1* and *P57*, genes encoding pro-proliferative and anti-proliferative factors, respectively, indicative of EED/BRD4 co-occupancy at these genes. Results together suggest a cooperativity between the two readers that bolsters each other's function; i.e., activation of *Ccnd1* and repression of *P57*. As cyclinD1 and P57 are core cell cycle regulators in essentially every cell type, our study may shed new light on broad pathophysiological processes.

RESULTS

Blocking EED's reader function mitigates angioplasty-induced IH in rat arteries

It was recently reported that the H3K27me3 writer EZH2 plays a positive role in the development of IH.[17–19] More recently, we found a genome-wide surge of H3K27me3 occupancy in IH-prone arteries.[20] The reader or functional effector of H3K27me3, EED, is known to enhance EZH2-catalyzed H3K27me3 deposition.[14,16] However, the role of EED in the disease context of IH remained unclear. Reports of discrete functions of EED and EZH2 indicate that the context-dependent action of EED cannot be simply extrapolated from that of EZH2.[4,8]

We thus determined the EED expression during neointima formation in a commonly used IH model of rat carotid artery balloon angioplasty.[11,21] We observed that EED markedly increased in injured arteries on post-angioplasty day 7 and remained high throughout day 14 (Figures 1A and 1B), mainly in the neointima and the media where the SMC marker αSMA is expressed (Figures 1C and S1). To further explore the human disease relevance of EED, we used patient samples of coronary arteries that contained neointima lesions at the edge of the implanted stent and control arteries without a stent (control samples). As illustrated in Figure 2, compared with controls, the cross-sections of stent-edge samples showed higher EED expression in the media and neointima (also see Figure S2). Therefore, the results from rat and human samples indicated that EED protein levels were elevated during neointima formation.

We were then interested to study the role of EED in IH using the model of angioplasty-injured rat carotid arteries. To block EED's reader function, we applied the first-in-class EED-selective inhibitor (EED226), which specifically blocks the H3K27me3-binding pocket in EED, thereby evicting EED from the nucleosome[16] and may also reduce EED protein levels as a secondary effect (Figure S3). The inhibitor dispersed in a thermosensitive hydrogel was administered around the arteries immediately after balloon injury. The injured arteries treated with EED226 or vehicle were collected on post-injury day 14 (Figure 3A). As indicated by morphometric analysis in Figure 3B, IH represented by the neointima/media (N/M) area ratio was 1.02 ± 0.152 in the control group and 0.411 ± 0.093 in the EED226-treated group. Thus, IH was reduced by ~60% in EED226-treated arteries compared with vehicle controls ($p = 0.008$). The lumen area increased from 1.990 ± 0.234 to 2.563 ± 0.141 ($p = 0.06$). There was no change in the length of external elastin lamina (EEL), which measures the overall vessel size, indicating a lack of undesirable vessel shrinkage. Given that the H3K27me3 level is a readout of EED's chromatin-associated function as aforementioned,[14,16] the H3K27me3 immunofluorescence in Figure 3C indicated a decrease of EED functional activity after treatment with EED226, hence validating the EED-targeting effect of this inhibitor. To our knowledge, these results represent the first demonstration of IH-mitigating efficacy of disrupting EED's H3K27me3-reader activity.

EED inhibition or knockdown attenuates SMC proliferation *in vitro*

It is well documented that post-injury neointima is formed principally due to SMC hyper-proliferation/migration, which is most potently stimulated by the mitogen PDGF-BB.[1,22] Thus, we next determined the specific role of EED in SMC proliferation and migration. As shown in Figure 4A, EED226 dose-dependently inhibited PDGF-stimulated SMC proliferation. This result was reproduced by using A-395,[23] another chemical inhibitor of EED's H3K27me3-reader activity that was developed in parallel to EED226.[16] Since EED226 at 25 μM was highly effective, we used this concentration throughout in the following experiments. Importantly, EED gene knockdown (Figure 4B) recapitulated the pharmacological outcome of attenuated SMC proliferation, thus supporting the specificity of the EED inhibitors. Consistently, treatment of SMCs with EED226 also impeded cell migration (Figure 4C) without changing SMC marker protein levels (Figure 4D). In accordance with the *in vivo* data in Figure 3C, the *in vitro* data from cultured SMCs indicated that EED226 reduced H3K27me3 levels (Figure 4D) detected on immunoblots (see the more pronounced effect after 72-h treatment in Figure S3), and EED gene knockdown had a similar effect (Figure 4E). Thus, these results confirm the EED-targeted functional specificity of the inhibitor drug EED226 and also implicate a chromatin-associated mechanism, based on the known EED role in facilitating H3K27me3 deposition.[15]

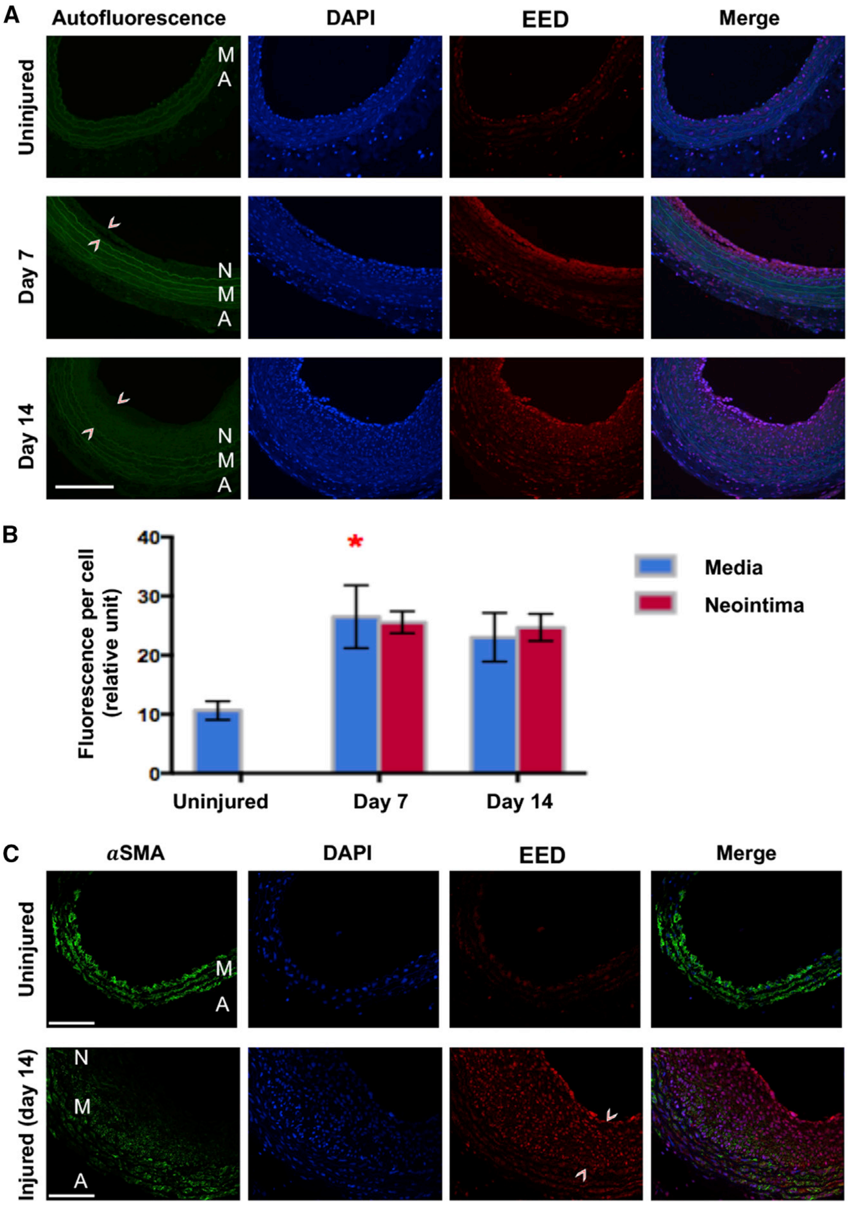

Figure 1. Upregulation of EED in rat arteries that undergo balloon angioplasty-induced IH

Angioplasty was performed in the rat common carotid artery. The contralateral artery in the same animal but without angioplasty served as the uninjured control. Animals were euthanized on post-angioplasty days 7 and 14 for preparation of artery cross-sections that were used for EED immunofluorescence. (A) Injury-induced increase of EED. Shown are representative images of EED immunofluorescence. Neointima is demarcated between arrowheads. The autofluorescence of elastic lamina profiles the medial layer. A, adventitia; M, media; N, neointima. Scale bar, 100 μm. (B) Quantification for (A). Fluorescence intensity and total DAPI-stained cells in each image field were measured by ImageJ. The data from five to six image fields of different cross-sections were pooled to generate the mean for each animal. The means from all animals in each group were then averaged, and the final mean (±SEM) was calculated. Statistics: ANOVA followed by Bonferroni test, $^*p < 0.05$ compared with the uninjured control; n = 5 rats (uninjured) or n = 3 rats (post-injury day 7, day 14). (C) Co-staining of EED and the SMC marker αSMA. Post-injury day-14 sections were used. Neointima is demarcated between arrowheads. A, adventitia; M, media; N, neointima. Scale bar, 100 μm.

Interestingly, EED knockdown also reduced cyclin D1 protein, a *bona fide* pro-proliferative cell cycle regulator (Figure 4E), and its mRNA (Figure 4F). Accordingly, EED overexpression enhanced H3K27me3 (Figure 4G), and also increased *Ccnd1* mRNA (Figure 4H). These *in vitro* results indicate that EED is a pro-proliferative factor in mitogen-stimulated SMCs, in keeping with its observed positive effects on neointima proliferation *in vivo* (Figure 3). Of particular interest, the loss- and gain-of-function results indicated that EED played a positive rather than negative role in *Ccnd1* transcription (Figures 4E–4H). This was unexpected since there was no prior evidence supporting a gene-activating role for EED, the well-known gene-repression H3K27me3 reader.[13]

BRD4 co-immunoprecipitates with EED and vice versa

We noted that the foregoing results of EED were reminiscent of that of the H3K27ac reader BRD4, a prominent pro-proliferative player in cancer cells[9] that also promotes SMC/neointima proliferation.[11,12] It was therefore motivating for us to pursue a functional link between these two epigenetic readers, whereas their association was not previously reported. The experiments led to yet another surprising finding. That is, overexpression of BRD4 led to a marked increase of H3K27me3 level (Figure 5A), reminiscent of EED's function of enhancing H3K27me3 deposition. This was counter-intuitive since BRD4 is not known for any methyltransferase activity[24,25] and the level of the H3K27me3 writer EZH2 was not altered by BRD4 overexpression (Figure 5B). On the other hand, EED and BRD4 both act as chromatin-associated scaffold proteins interacting with a variety of proteins.[16,25] In this light, a plausible hypothesis would be that these two readers cooperate to support each other's function, namely, H3K27me3 enhancement and *Ccnd1* transcription, respectively. We thus performed coIP experiments to detect their association. Indeed, we observed robust BRD4 coIP with EED, and vice versa (Figures 5C–5F). The EED protein contains a flexible N terminus (residues 1–80) and seven copies of the WD-repeat motif forming a seven-bladed propeller structure.[26] Our coIP analysis suggested that it was the N terminus that mainly contributed to the EED/BRD4 interaction. As seen in

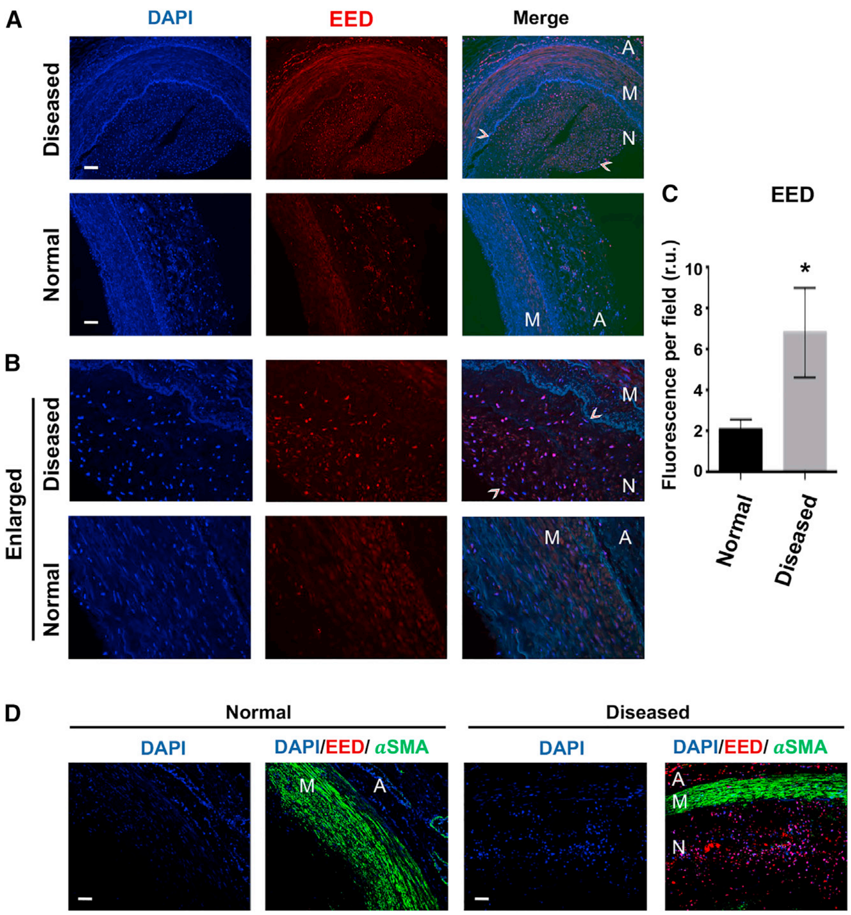

Figure 2. Upregulation of EED in the neointima of human coronary artery samples

Human coronary artery samples were purchased from CVPath Institute (Gaithersburg, MD). Diseased, refers to cross-sections from the edge of the implanted stent; Normal refers to cross-sections from coronary arteries without a stent. Scale bar, 50 μm. (A) Representative images of EED immunofluorescence (10×). Neointima is demarcated between arrowheads. A, adventitia; M, media; N, neointima. (B) Enlarged view to discern immunostained individual nuclei in the neointima. (C) Quantification: data are presented as mean ± SD of fluorescence intensity per high-power field (including neointima and media) from different cross-sections (n = 4 patients). Statistics: unpaired Student's t test, *$p < 0.05$. (D) Co-staining of EED and the SMC marker αSMA. A, adventitia; M, media; N, neointima.

Figures 5G/5H, each of the EED constructs that contained the N terminus effectively immunoprecipitated endogenous BRD4 protein but the N terminus-truncated protein (WD1-7) did not. Furthermore, supporting the relevance of the EED/BRD4 complex to their respective chromatin-associated H3K27me3 or H3K27ac reader function, either a blocker of the EED/H3K27me3 interaction (EED226) or BRD4/H3K27ac interaction (JQ1) disrupted the BRD4 coIP with EED (Figures 5I/5J). It is generally thought that BRD4 as an H3K27ac reader prompts gene activation whereas EED as an H3K27me3 reader reinforces gene repression.[13,24] It is therefore intriguing to observe that BRD4 and EED, previously known as playing opposing roles in the epigenetic control of transcription, are physically associated with each other.

EED and BRD4 co-occupy at both pro-proliferative Ccnd1 and anti-proliferative P57

Cyclin D1 is a potent cell cycle activator that prompts proliferation/migration, whereas p57 is a *bona fide* cell cycle inhibitor.[22] *Ccnd1* is activated by H3K27ac, while *P57* is repressed by H3K27me3.[20,27] Interestingly, *Ccnd1* and *P57* reside on the same chromosome (mouse, *Chr7*; human, *Chr11*), suggestive of possible three-dimensional proximity. However, whether H3K27me3 reader EED and H3K27ac reader BRD4 co-occupy at these genes was not previously known. To determine the functional significance of the observed EED/BRD4 protein complex (Figure 5), we performed chromatin IP (ChIP)-qPCR experiments (Figure 6) to determine EED and BRD4 occupancy at *Ccnd1* and *P57*.

Interestingly, as indicated in Figure 6A, ChIP with either an EED-specific antibody or BRD4-specific antibody (binding the respective endogenous protein) pulled down both *Ccnd1* and *P57* promoter DNA fragments in SMCs treated with PDGF-BB. Moreover, PDGF-stimulated pulldown of *Ccnd1* and *P57* DNA was disrupted by either EED226 or JQ1 (Figure 6A). For negative control, ChIP-qPCR with primers targeting a distal region of the *P57* promoter did not show PDGF-responsive and EED/BRD4 inhibitor-sensitive changes (Figure S4), indicative of site-specific genomic occupancy of EED and BRD4. Thus, the ChIP-qPCR data, concurring with the EED/BRD4 coIP data, indicates that EED and BRD4 co-occupy at *Ccnd1* and *P57*. While this result represents the first observation involving both EED and BRD4, it could be plausibly rationalized by the working model schematized in Figure 6B. That is, as BRD4 and EED are in the same chromatin-associated complex, they are readily cross-linked together, and IP with a BRD4 antibody should pull down not only *Ccnd1* promoter DNA but also that of *P57*, and so should IP with an EED antibody. Even though the time course experiment indicated synchronized EED and BRD4 protein level changes in response to PDGF-BB stimulation of SMCs (Figure S5), whether these two proteins bind to the promoter DNA simultaneously remains unclear. Importantly, we were able to verify the functional significance of the observed EED/BRD4 co-occupancy. As demonstrated in Figure 6C, treatment with PDGF-BB activated *Ccnd1* and repressed *P57*, consistent with the known respective functions of BRD4 and EED. More interestingly, inhibition of either EED or

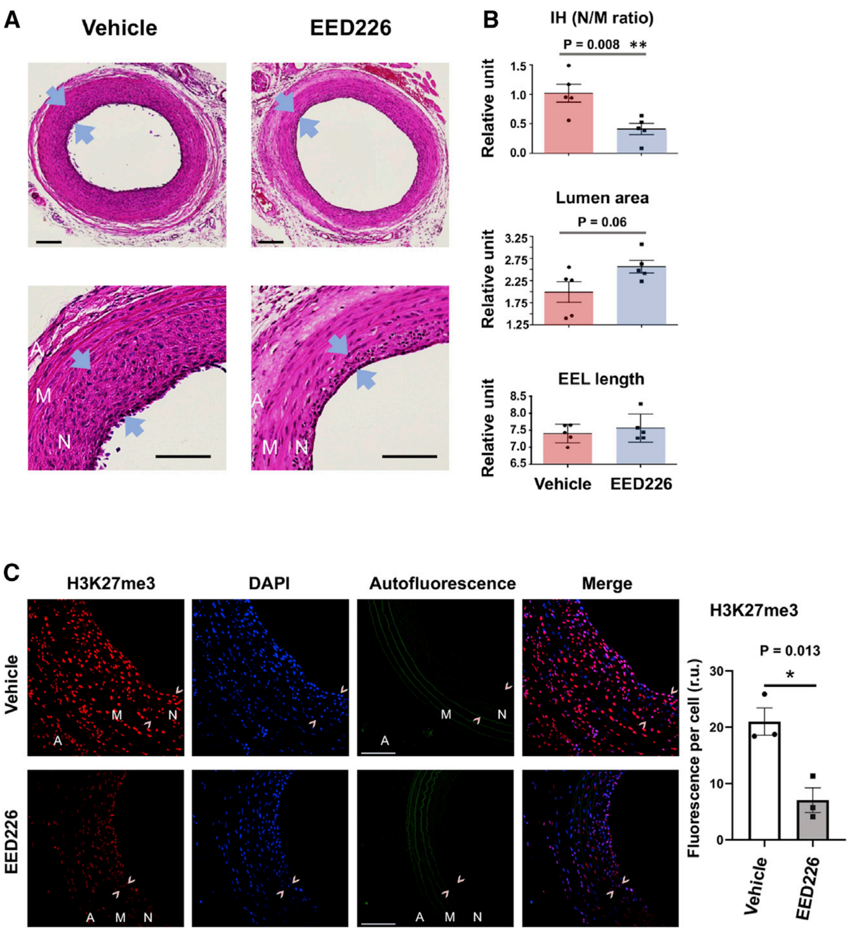

Figure 3. Selective EED inhibitor reduces IH and H3K27me3 in balloon-injured rat carotid arteries
IH-inducing balloon angioplasty was performed in rat common carotid arteries followed by perivascular administration of the EED-selective inhibitor EED226. Cross-sections were prepared from the arteries collected on day 14 post injury and H&E stained. IH is measured as the N/M area ratio. Neointima is demarcated between arrowheads. A, adventitia; M, media; N, neointima. Scale bar, 100 μm. (A and B) Morphometric analysis. For quantification, the data from sections cut at different sites of the injured artery were pooled to generate the mean for each animal. The means from all animals in each treatment group were then averaged, and the final mean ± SEM was calculated. Statistics: unpaired two-tailed Student's t test, $^{**}p < 0.01$, n = 5 animals. (C) Immunostaining of H3K27me3. Cross-sections prepared from the arteries collected on post-injury day 14 were immunostained for H3K27me3, a readout of EED function. Neointima is demarcated between arrowheads. A, adventitia; M, media; N, neointima. Scale bar, 100 μm. Quantification: The immunofluorescence intensities from sections cut at different sites of the injured artery were pooled to generate the mean for each animal (five or six sections per rat). The means from all animals in each treatment group were then averaged, and the final mean ± SEM was calculated. Statistics, unpaired two-tailed Student's t test, $^{**}p < 0.01$, n = 3 animals.

BRD4 (with EED226 or JQ1) abolished PDGF-induced changes, in both *Ccnd1* and *P57* mRNA levels, indicative of an EED/BRD4 functional cooperativity. Furthermore, considering that JQ1 is a pan-inhibitor selective to the bromodomain and extra terminal (BET) family,[28] we silenced the BET family members BRD2, BRD3, and BRD4 individually in SMCs with their specific shRNAs.[11,29] The immunoblot data indicated that although silencing either BRD2 or BRD4 reduced the levels of H3K27me3 and cyclin D1, only BRD4 silencing increased P57 protein, and BRD3 silencing did not show an effect (Figure 6D). This result is consistent with the specific role of BRD4 in the EED/BRD4 functional cooperativity.

EED co-immunoprecipitates with STAT3 and supports STAT3 activation

A prominent pattern whereby epigenetic readers enact transcriptional regulations is their binding with context-specific transcription factors at select gene loci.[5,9] While our data demonstrated a role for EED in regulating SMC/neointima proliferation (Figures 1, 2, 3, 4 and 5), the transcription factor that EED may partner with was not known. Recent reports lent useful hints. For instance, it is well documented that activation of signal transducer and activator of transcription 3 (STAT3) prompts SMC and neointima proliferation (i.e., IH),[30,31] and JQ1 blocks STAT3 activation in murine glioma stem cells.[32] We were therefore motivated to explore a possible EED/STAT3 association. As shown in Figures 7A and 7B, while phosphorylated STAT3 increased in SMCs after PDGF-BB stimulation, it was concentration-dependently inhibited by JQ1 which disrupts the BRD4/H3K27ac interaction.[9] Remarkably, PDGF-stimulated STAT3 activation (phosphorylation at Tyr705)[33] was also concentration-dependently inhibited by EED226, which blocks the EED binding with H3K27me3.[16] We thus next determined a possible EED/STAT3 interaction via coIP experiments. The data in Figures 7C and 7D showed a strong STAT3 coIP with EED (30-fold over background control). Moreover, this result was confirmed by EED coIP with STAT3 (Figures 7E and 7F). Thus, the robust reciprocal coIP between EED and STAT3 supports their physical association (direct or indirect), for which the EED N terminus appears to be required (Figure S6). We further validated the functional significance of this EED/STAT3 association by silencing STAT3 in SMCs, which nearly abolished PDGF-stimulated expression of the known target gene *Ccnd1*[31] at both mRNA and protein levels (Figures 7G and 7H). Therefore, the observed EED/STAT3 interaction is consistent with the foregoing result of EED positively regulating *Ccnd1* in proliferating SMCs (Figure 4). Since there are other transcription factors that are predicted to bind to the *Ccnd1* or *P57* promoter,[34,35] whether EED interacts with some of them (Figure S7) in the context of SMC proliferation requires future research to elucidate.

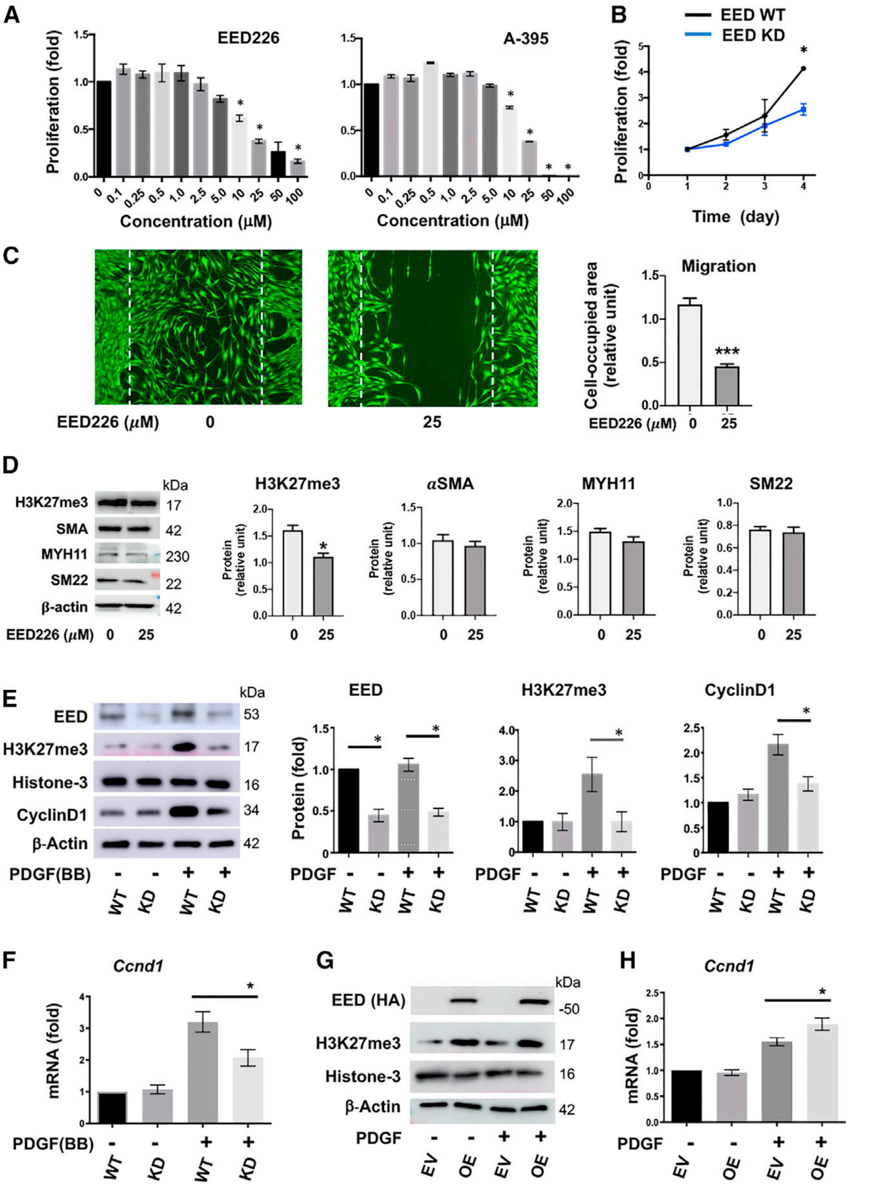

Figure 4. Nullifying EED hampers vascular SMC proliferation; EED positively regulates *Ccnd1* expression

MOVAS cells were starved overnight in basal medium (containing 0.5% FBS) prior to adding 20 ng/mL PDGF-BB (or solvent control) to stimulate cell proliferation. EED226 or A-395 of increasing concentrations (or an equal amount of DMSO) was pre-incubated with the cells for 4 h prior to PDGF stimulation. For EED knockdown (KD), the cells were transduced with a lentivector to express EED-targeting sgRNAs and the Cas9 protein. Quantification: western blot densitometry was first normalized to loading control (β-actin) and then to the basal condition without PDGF stimulation (the first bar in each plot). The data values from three to four independent repeat experiments were averaged to calculate mean ± SEM (n = 3–4). Statistics, one-way analysis of variance (ANOVA) followed by Bonferroni *post hoc* test, *$p < 0.05$ (compared with the no-inhibitor condition in A). (A) Effect of EED inhibition on SMC proliferation. CellTiter-Glo assay was carried out at 72 h after PDGF(-BB) stimulation. *$p < 0.05$, compared with vehicle control. (B) Effect of EED knockdown on SMC proliferation (CellTiter-Glo assay). (C) Effect of EED inhibition on SMC migration (scratch assay). Primary rat aortic SMCs were cultured to ~90% confluency, pre-incubated with zero (equal amount of DMSO) or 25 μM EED226 for 2 h, scratched, and then incubated with fresh medium for 24 h. White dashed lines demarcate the original scratched, cell-free area. The area reoccupied by cells was used to measure migration. Student's t test, ***$p < 0.001$. (D) Effect of EED inhibition on SMC marker protein expression. Starved primary rat aortic SMCs were incubated with zero (equal amount of DMSO) or 25 μM EED226 for 24 h. Student's t test, *$p < 0.05$. (E) Effect of EED knockdown (verified by western blot) on cyclinD1 protein levels. Cells were harvested at 24 h after PDGF(-BB) stimulation. (F) Effect of EED knockdown on cyclinD1 mRNA levels. Cells were harvested at 24 h after PDGF(-BB) stimulation. (G) EED overexpression verified by western blot. Note increased H3K27me3. (H) Effect of EED overexpression on cyclinD1 mRNA levels. qRT-PCR reading was first normalized to *Gapdh* using the delta-delta ct method, and then to the non-stimulated basal condition (the first bar in each plot). Mean ± SEM was calculated by averaging the data from three independent repeat experiments (n = 3).

EED inhibition dampens STAT3 activation and reduces cyclinD1 in injured rat arteries

To determine the effect of EED inhibition on STAT3 activation *in vivo*, we immunostained phospho-STAT3 and its target gene product cyclinD1 using cross-sections of angioplasty-injured rat carotid arteries treated with the EED inhibitor (EED226) or vehicle control. Indeed, the number of P-STAT3-positive cells (normalized to total cells) was lower in the EED226 (versus vehicle)-treated animal group (31.33 ± 5.487 versus 66.33 ± 5.696, $p < 0.05$), particularly in the medial and neointimal layers that are mainly composed of SMCs (Figure 8). Consistent with the *in vitro* data supporting a role for EED in enhancing *Ccnd1* activation, cyclinD1, which is a known STAT3 target gene product, was remarkably reduced in the EED226-treated group compared with vehicle controls (6.0 ± 1.225 versus 14.50 ± 1.50, $p < 0.01$). Thus, these results provide the first *in vivo* evidence for the EED/STAT3-cyclinD1 axis in injured arteries.

DISCUSSION

SMC proliferation perpetuates IH in the vascular wall, a common etiology that ultimately leads to adverse cardiovascular conditions.[1] While implanted drug-eluting stents can reduce IH, knowledge deficit in epigenetic regulations of SMC responses has limited precise therapeutic targeting. Histone modifications H3K27ac and H3K27me3 render locally opened and closed chromatin states, respectively, corresponding to gene activation and repression.[4] Pro-proliferative gene activation has long been regarded as the predominant event that drives SMC and neointima

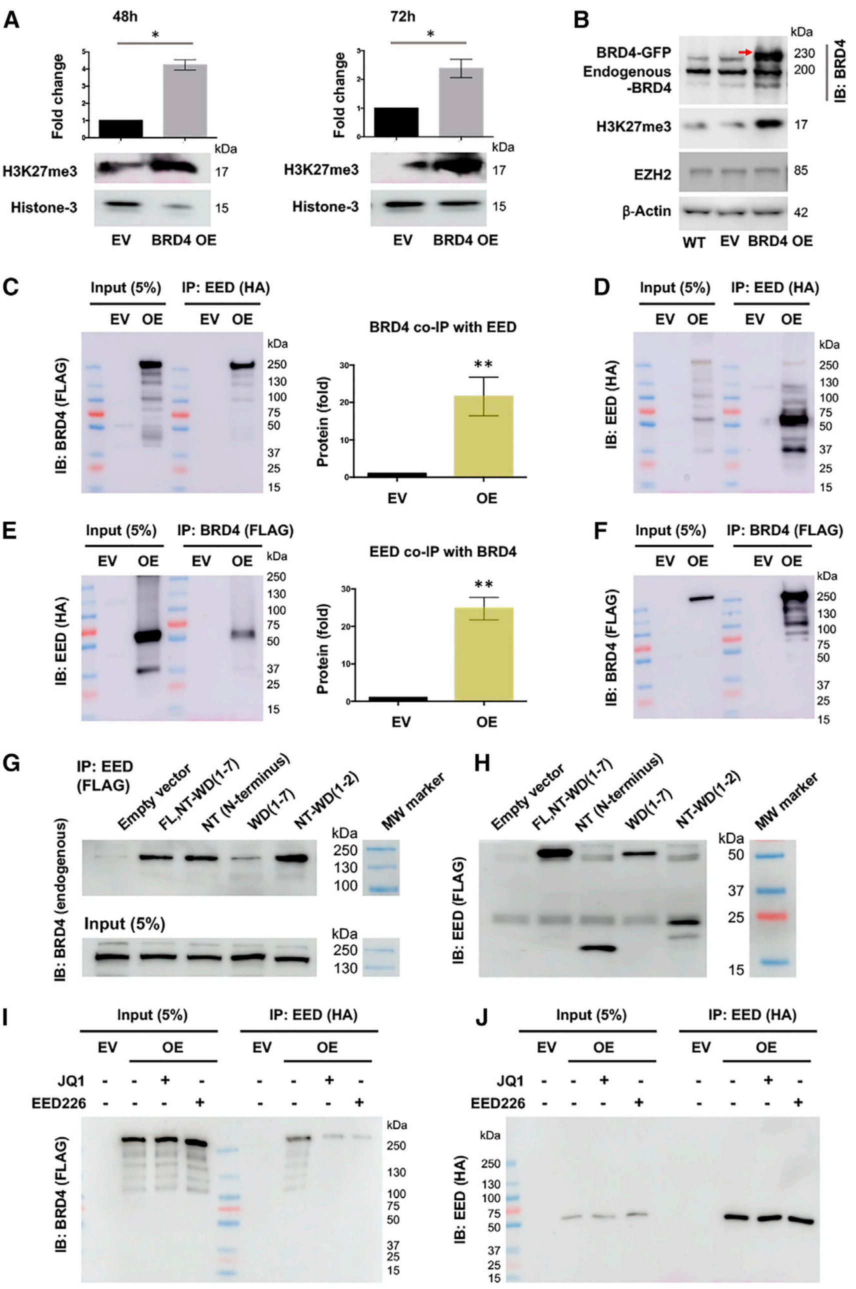

Figure 5. Reciprocal coIP of EED and BRD4

(A) Increased H3K27me3 deposition due to BRD4 overexpression. Cells (HEK293A) were transfected with plasmids, as detailed in section "materials and methods," and harvested at 48h or 72 h for western blot analysis. EV, empty vector; OE, overexpression. H3K27me3 was normalized to histone-3. Student's t test, $*p < 0.05$, $n = 3$ independent repeat experiments. (B) No change in the EZH2 protein level after BRD4 overexpression. Shown is one of two similar experiments. Red arrow indicates the overexpressed BRD4 band distinct from the non-specific upper band in the EV and wild-type (WT) lanes. (C and E) Reciprocal BRD4 and EED coIP. Cells were transfected to express HA-tagged EED or FLAG-tagged BRD4. An anti-HA or anti-FLAG antibody was used for IP or the corresponding immunoblotting (IB). EV, empty vector. Student's t test, $**p < 0.01$, $n = 4$ independent repeat experiments. (D and F) Western blots showing effective IP of EED or BRD4. The protein samples were from the same experiments as in (C) and (E). Each blot is representative of four repeat experiments. (G and H) CoIP of full-length or truncated EED proteins with endogenous BRD4. FLAG-tagged EED constructs were expressed in HEK293A cells, and an anti-FLAG antibody was used for IP. An antibody for endogenous BRD4 was used for IB. FL, full-length, including the N terminus (NT) and seven WD repeats. The two faint bands in (H) should be IgG heavy chain and light chain based on their sizes. (I and J) Sensitivity of the BRD4/EED coIP to their respective inhibitors. Note that the inhibitors did not alter BRD4 protein levels in the input, nor the IP of EED with the anti-HA antibody.

proliferation.[1,22] In contrast, EED is canonically known as a gene represser[14] rather than an activator, and its role in IH was not previously determined. We report here for the first time a role for EED in promoting cyclinD1 gene (*Ccnd1*) activation in the context of SMC/neointima proliferation. We further provide evidence that this novel EED function likely occurs via its cooperativity with BRD4, a gene-activating H3K27ac reader.[9]

We were initially intrigued by our inadvertent finding of EED supporting *Ccnd1* activation. This was a surprise because EED is well known as a reader of H3K27me3, a histone mark of gene repression.[16] In our attempt to interpret this conundrum, we noticed that the positive role of EED in SMC/neointima proliferation resembled that of BRD4.[11] This appeared paradoxical, given that EED and BRD4 are traditionally regarded as functioning in opposite directions: gene repression and activation, respectively.[5,9,14] Along this line, it is also a perplexing observation that BRD4 overexpression mirrored EED's function of enhancing H3K27me3 deposition. Apparently, H3K27me3 deposition was not catalyzed by BRD4, since such a BRD4 enzymatic activity is not known to exist, although BRD4's intrinsic acetyltransferase activity has been reported.[24] Enhanced H3K27me3 was not caused by increased H3K27me3 writer EZH2 either, as its protein level was not changed by BRD4 overexpression, and we did not detect a BRD4/EZH2 interaction in coIP experiments (Figure S8).

To reconcile these results, we inferred that EED and BRD4 may have functioned cooperatively, based on the following rationales. First, like EED, BRD4 is known as a chromatin-associated scaffold protein capable of interacting with multiple cofactors.[25,36] In fact, although previously

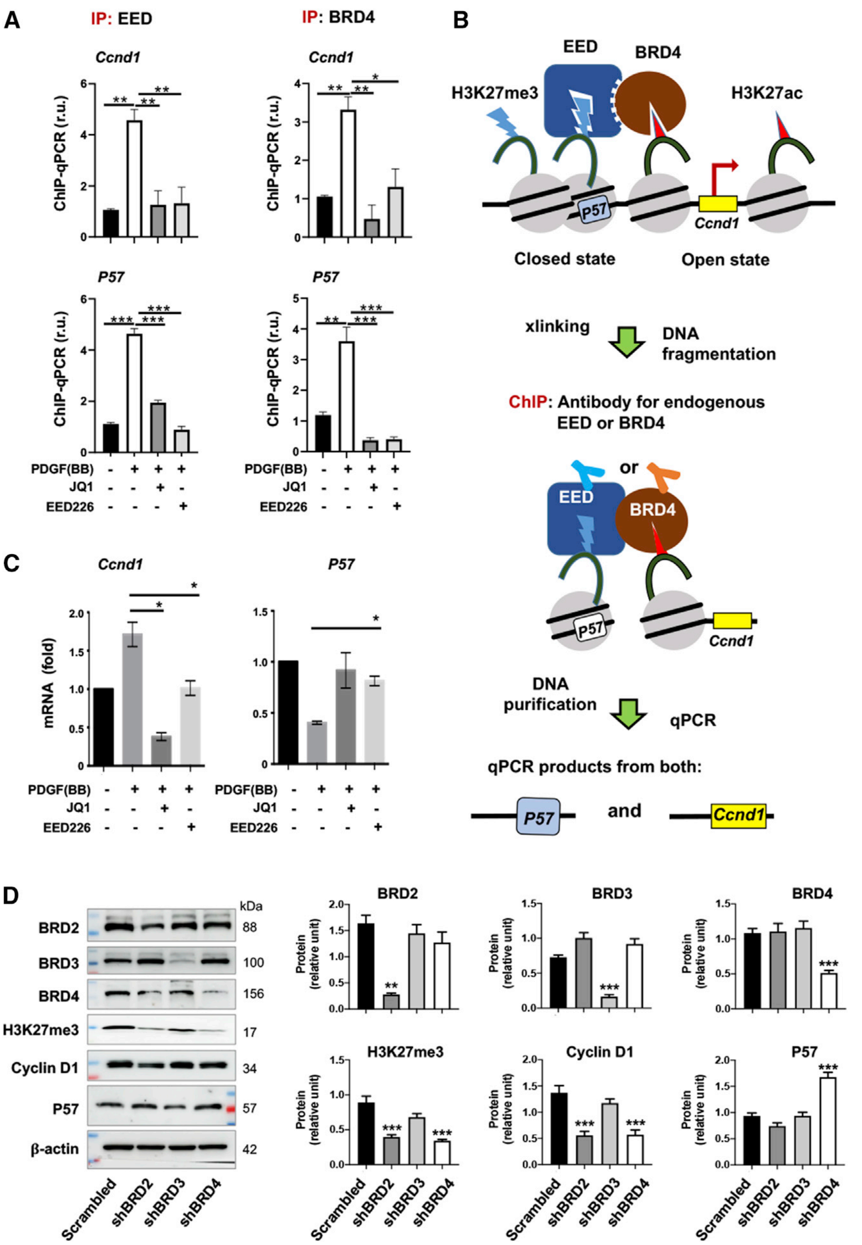

Figure 6. ChIP-qPCR indicative of co-occupancy of EED and BRD4 at *Ccnd1* and *P57*

MOVAS cells were cultured, starved, pre-incubated with EED226 or JQ1 (1 μM), and then stimulated with PDGF-BB, as described for Figure 4. Cells were harvested for assays after 48-h PDGF stimulation. ChIP was performed using an antibody specific for endogenous EED or endogenous BRD4. (A) ChIP-qPCR. ChIP was performed using an antibody specific for endogenous EED or endogenous BRD4. Quantification: three independent ChIP experiments were performed at separate times (n = 3). The qPCR reading was first normalized to *Gapdh* using the delta-delta ct method, and then to the basal-condition (no PDGF-BB, no inhibitor) value from one of the three repeat experiments. All readings were thereby converted to fold changes and averaged to produce mean ± SEM. Statistics: one-way ANOVA and Bonferroni *post hoc* test, $*p < 0.05$, $**p < 0.01$, $***p < 0.001$. (B) Schematic working model of EED and BRD4 co-occupancy. H3K27me3 as a gene-repressing mark is associated with a closed chromatin state (note condensed nucleosomes and repressed *P57*), H3K27ac as a gene-activating mark (e.g., at *Ccnd1*) is associated with an open chromatin state. The dashed line between EED and BRD4 depicts a possibility of these two proteins in the same complex but not necessarily contacting each other. Due to proximity (or interaction), EED and BRD4 as well as the respective nucleosomes they bind to can be cross-linked together. After DNA fragmentation, the remaining DNA regions of *Ccnd1* and *P57* corresponding to BRD4 and EED co-occupancy can thus be pulled down via ChIP and detected via qPCR. (C) Either EED226 or JQ1 can avert PDGF-induced changes in *Ccnd1* and *P57* mRNA levels. Quantification: qRT-PCR reading was first normalized to *Gapdh* using the delta-delta ct method, and then to the non-stimulated basal condition (first bar in each plot). Mean ± SEM was calculated by averaging the data from three independent repeat experiments (n = 3). Statistics: one-way ANOVA and Bonferroni *post hoc* test, $*p < 0.05$. (D) BRD4 silencing but not BRD2 or BRD3 silencing increases P57 protein. Rat primary SMCs were infected with lentivirus to express scrambled or BRD-specific shRNA. Data are presented as mean ± SEM, n = 3 independent repeat experiments. Statistics: one-way ANOVA and Tukey test, $*p < 0.01$, $***p < 0.001$ (compared with the first bar).

thought to be global regulators, EED and BRD4 have each recently been shown to confer specific regulations by complexing with combinatorial sets of binding partners at specific genomic loci.[7,9] Second, in keeping with literature evidence that EED and BRD4 each occupies at *P57* and *Ccnd1*, respectively[27] (though an EED/BRD4 interaction was not known), *Ccnd1* and *P57* reside in the same chromosome, suggesting possible three-dimensional proximity. Indeed, as a prominent feature of chromatin 3D architecture, even genes in remote genomic regions could be pulled together (e.g., through looping).[37] Thus, the duo of EED/BRD4 seemed probable, which could respectively bind with their cognate mark (H3K27me3, H3K27ac) at neighboring *P57* and *Ccnd1*. Indeed, the coIP data indicated an EED/BRD4 interaction, which could be mediated either by direct protein contacts or by a yet-to-be-identified co-factor. To the best of our knowledge, a histone mark-associated EED/BRD4 interaction has not been previously reported, although consistent evidence exists indicating improved tumor treatment efficacy through combined inhibitors of EZH2 and BRD4.[38] Furthermore, via ChIP-qPCR, we confirmed the EED/BRD4 association on chromatin at their target genes *P57* and *Ccnd1*. That is, ChIP with either an anti-EED or anti-BRD4 antibody was able to pull down both *P57* and *Ccnd1* DNA fragments. Importantly, the EED/BRD4 co-occupancy at *P57* and *Ccnd1* was closely associated with their reader functions, as evident

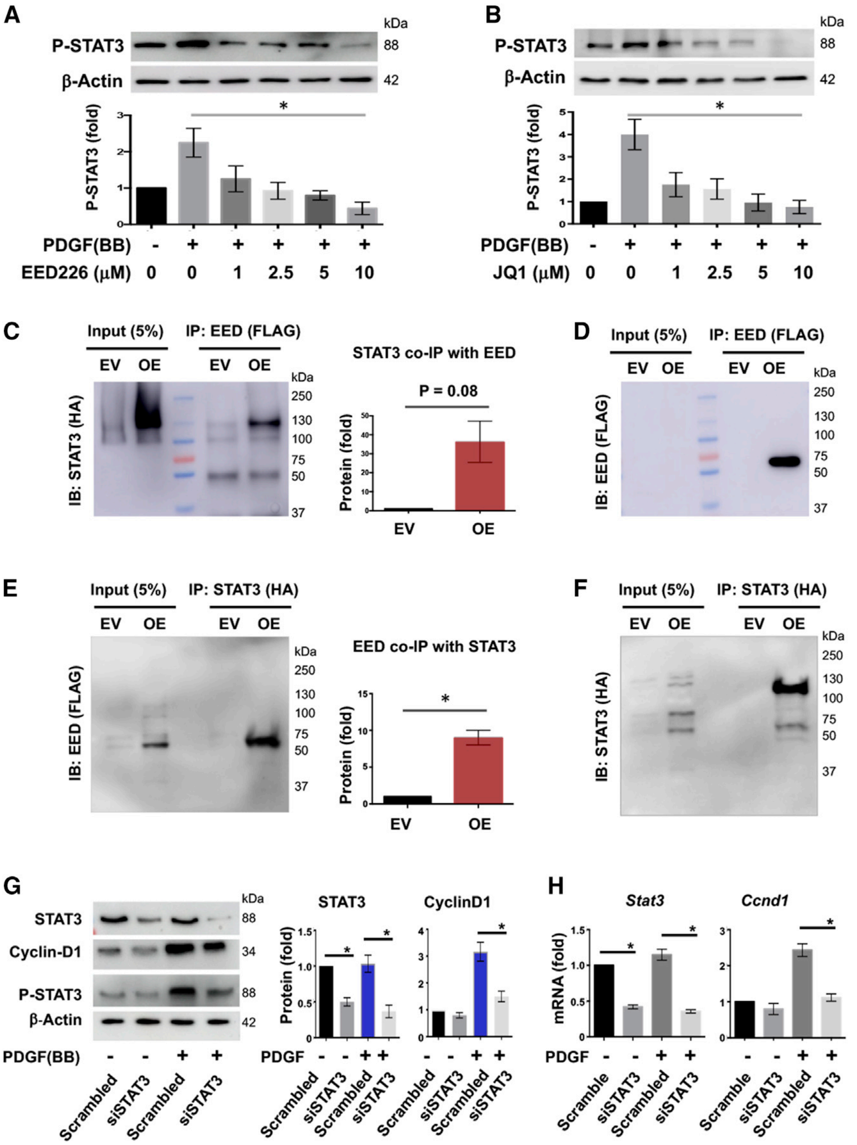

Figure 7. An EED/STAT3 axis that regulates downstream *Ccnd1* expression

(A and B) Either EED inhibition or BRD4 inhibition blocks STAT3 activation (phosphorylation at Tyr705). MOVAS cells were cultured, starved, pre-incubated with vehicle (DMSO) or increasing concentrations of EED226 or JQ1 for 4 h, and then stimulated with PDGF-BB, as described for Figure 4. The cells stimulated with PDGF (BB, 15 min) were used for western blot analysis of phospho-STAT3. Western blots were quantified as described for Figure 4. One-way ANOVA followed by Bonferroni *post hoc* test, $*p < 0.05$ (second versus last bar), n = 4 independent repeat experiments. (C and E) Reciprocal EED and STAT3 coIP. Cells were transfected with plasmids, as detailed in section "materials and methods." An anti-FLAG or anti-HA antibody was used for IP or subsequent IB. EV, empty vector; OE, overexpression. Student's t test, $*p < 0.05$; n = 4 independent repeat experiments. (D and F) IB indicating effective IP of EED or STAT3. The protein samples were from the same experiments as in (C) and (E). Each blot is representative of four repeat experiments. (G and H) Regulation of cyclinD1 expression by STAT3. MOVAS cells were transduced with lentivirus to express scrambled or STAT3-specific shRNA for 12 h in full medium. After recovery in fresh medium for 12 h, the cells were starved overnight and then stimulated with PDGF(-BB) for 24 h prior to use for analysis. Western blot and RT-qPCR data were quantified as described for Figure 4. One-way ANOVA followed by Bonferroni *post hoc* test, $*p < 0.05$, n = 3 independent repeat experiments.

from the way ChIP-qPCR results could be averted by either disrupting EED/H3K27me3 or BRD4/H3K27ac with an inhibitor. In accordance, in our recent report,[29] ChIP sequencing using an H3K27me3 antibody and angioplasty-injured rat artery tissues demonstrated a prominent H3K27me3 enrichment at *P57*, further supporting a chromatin association of EED's function as an H3K27me3 reader.

Taken together, while EED is canonically known for gene repression,[13,14] herein it is shown to enhance *Ccnd1* activation; similarly, BRD4 is a potent transcription co-activator[39] but is shown here to repress *P57*. The "crossover" functions of the two histone code readers could be plausibly reconciled in a working hypothesis; i.e., EED and BRD4 co-occupy neighboring nucleosomes, whereby EED binding with H3K27me3 represses *p57* and BRD4 binding with H3K27ac activates *Ccnd1* (see schematic in Figure 6B). As such, this chromatin-associated assembly may be stabilized by the interaction of EED and BRD4, which thereby co-opt and support each other's reader function to repress anti-proliferative *P57* while activating pro-proliferative *Ccnd1*. Future research is warranted to further examine this hypothesis (for example, to extrapolate our finding of the EED/BRD4 cooperation to genes beyond *Ccnd1* and *P57*).

Nevertheless, the observed EED/BRD4 cooperativity is consistent with our *in vitro* and *in vivo* functional studies. Specifically, while *Ccnd1* was activated and *P57* was repressed, both occurred in PDGF-stimulated SMCs, and pre-treatment with either an EED or BRD4 inhibitor could avert both events and stymie SMC proliferation and IH (regarding BRD4 and IH, see our report[11]). Downstream, STAT3 appeared to be an important transcription factor that mediated EED's function, in keeping with its established role in promoting SMC/neointima proliferation.[31] As indicated by our data, while STAT3 silencing abrogated PDGF-stimulated *Ccnd1* activation in SMCs, EED inhibition suppressed STAT3 activation *in vitro* and *in vivo* in injured arteries that underwent IH, and also reduced cyclinD1 expression *in vitro* and *in vivo*. In line with our results, it was recently reported that pharmacological disruption of EED function inhibited STAT3 activation in prostate cancer cells.[40] However, an EED/STAT3 interaction has not previously been described.

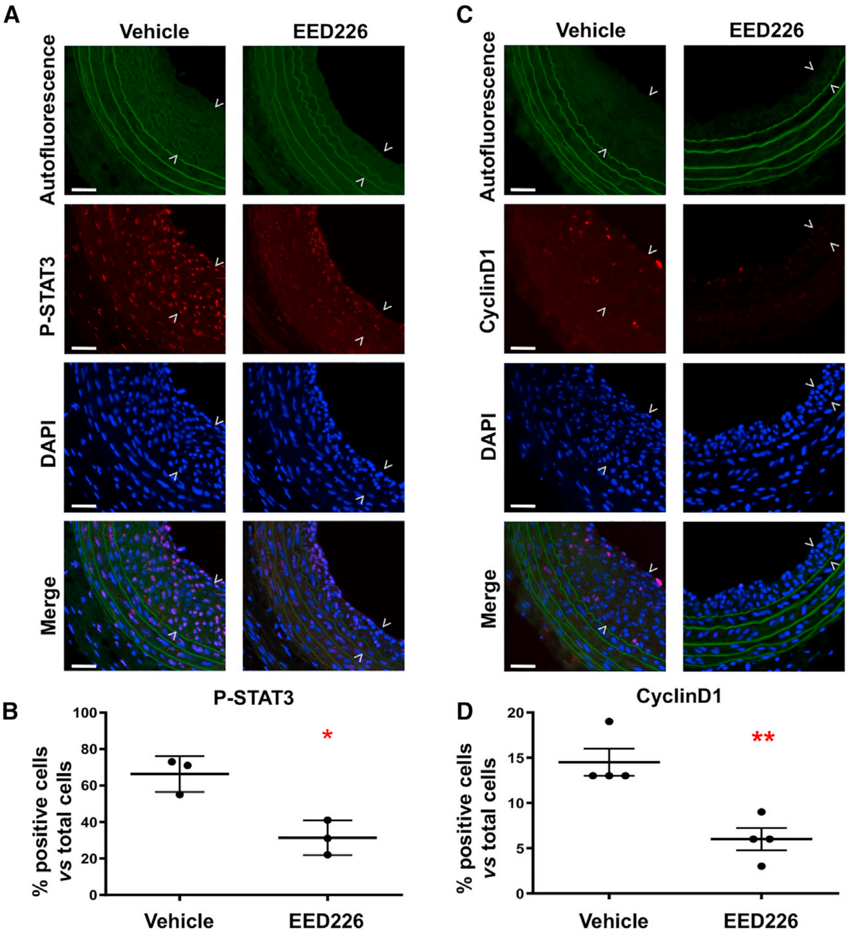

Figure 8. EED inhibition attenuates STAT3 phosphorylation and cyclinD1 expression in balloon-injured rat arteries

IH-inducing balloon angioplasty was performed in rat common carotid arteries followed by perivascular administration of EED inhibitor (EED226). Cross-sections of arteries collected on post-angioplasty day 14 were used for immunofluorescence staining. Data quantification: positively stained cells and total DAPI-stained cells in each image field were manually counted. The data from five to six image fields of different cross-sections were pooled to generate the mean for each animal. The means from all animals in each of the vehicle- or EED226-treated animal groups were then averaged, and the final mean (±SEM) was calculated. (A) Representative P-STAT3 immunofluorescence images. Neointima is indicated between arrowheads. Scale bar, 50 μm. The autofluorescence of elastic lamina profiles the medial layer. (B) Quantified P-STAT3 immunostaining. Statistics: unpaired Student's t test, $^{*}p < 0.05$, n = 3 animals. (C) Representative cyclinD1 immunofluorescence images. Neointima is indicated between arrowheads. Scale bar, 50 μm. The autofluorescence of elastic lamina profiles the medial layer. (D) Quantified cyclinD1 immunostaining. Statistics: unpaired Student's t test, $^{**}p < 0.01$, n = 4 animals.

Conclusions

EED is an H3K27me3 reader with an established gene-repressing function. However, herein we found EED promoted the gene activation of the pivotal pro-proliferative factor cyclinD1. This likely occurred through its chromatin-associated cooperativity with gene-activating BRD4. Of interventional significance, EED inhibition mitigated IH. Recently, selective inhibitors have been successfully developed for EED, BRD4, and other epigenetic players.[5,41] This progress has fueled considerable enthusiasm for epigenetic targeting for therapeutic purposes, with some inhibitors rapidly advancing into clinical trials for cancer and other conditions.[10,15] On the other hand, the mechanistic cooperativity of histone mark readers remains elusive overall.[38] We envision that future research to elucidate novel regulatory mechanisms of EED and BRD4 in proliferative diseases will extend our understanding of dysregulated chromatin states and help guide precision interventions.

MATERIALS AND METHODS

Materials

Main resources, including cells, reagents, kits, and other materials, are listed in Table S1 unless otherwise specified below. Human coronary artery samples were purchased from CVPath Institute (Gaithersburg, MD.

Animals

All animal studies conform to the Guide for the Care and Use of Laboratory Animals (National Institutes of Health, 2011, eighth edition). The study protocol was approved by the Animal Care and Use Committee at the University of Virginia.

IH model of rat carotid artery balloon angioplasty

We performed balloon angioplasty to induce IH, following the procedures detailed in our previous reports.[11,29] To minimize variables, we used only male Sprague-Dawley rats (Charles River Laboratories, Wilmington, MA) with similar body weights (330–350 g), and the surgery was performed by the same person throughout; morphometric analysis was performed by a student blinded to the assignment of animal groups. Throughout the procedure, rats were kept anesthetized via inhalation of 2%–2.5% isoflurane at a flow rate of 2 L/min. After dissecting the left common carotid artery, we inserted a 2-F balloon catheter (Edwards Lifesciences, Irvine, CA) from an arteriotomy on the left external carotid artery, and inflated it to 1.5 atm. The balloon was slowly withdrawn to the carotid bifurcation, where it was deflated and re-inserted. The insertion and withdrawal were repeated for four rounds, with the last one performed by slightly rotating the balloon while withdrawing. The inflated balloon injured the artery wall not only by overstretching but also by endothelial denudation, thereby inducing IH. After angioplasty (and inhibitor administration, see below), blood flow was resumed and the neck incision was closed. The animal was kept on a 37°C warm pad to recover. For postoperative analgesia, carprofen (5 mg/kg, every 24 h for a total of 72 h) was administered systemically, and bupivacaine (0.25%) was given through intra-incisional injection prior to skin closure.

Perivascular administration of the EED-selective inhibitor EED226

EED226 is a selective EED inhibitor that recently became available.[16] The inhibitor dissolved in DMSO (vehicle) was periadventitially administered immediately following balloon angioplasty, as described in our previous report.[11] Briefly, the stocks of thermosensitive AK12 hydrogel (Akina, Indianapolis, IN) and Pluronic gel (Sigma-Aldrich, St. Louis, MO) each was prepared by shaking in the cold room for ~3 days to completely dissolve in PBS. The two gels were mixed in a 1:1 ratio and added with EED226 from a cold DMSO stock solution. The EED226/gel mixture was then applied to the periadventitial space of the injured artery, which at body temperature underwent phase transition to a semisolid state. For each animal, a total 10 mg of EED226 dispersed in 400 μL of hydrogel was administered. The surgery of the angioplasty model was then completed as described above.

Morphometric analysis of IH

At 14 days after angioplasty, the injured common carotid arteries, treated either with EED226 or its vehicle control (equal amount of DMSO), were harvested from anesthetized animals following perfusion fixation at a physiological pressure of 100 mm Hg. The animals were immediately euthanized in a chamber gradually filled with CO_2. Cross-sections were prepared from the artery samples and stained with H&E, and planimetric parameters were measured and calculated.[11] These included EEL area (inside external elastic lamina), IEL area (inside internal elastic lamina), lumen area, intima area (= IEL area − lumen area), and media area (= EEL area – IEL area). IH was calculated as the intima/media area ratio. ImageJ was used for all measurements by a student blinded to treatment groups. The data from three to five sections were pooled to generate the mean for each animal. The means from the whole animal group were then averaged, and the standard error of the mean (SEM) was calculated.

Vascular SMC culture and EED knockdown

SMCs (MOVAS cell line from ATCC) were cultured in DMEM supplemented with 10% fetal bovine serum (FBS) and 100 U/mL penicillin-streptomycin with 5% CO_2 at 37°C under humidified conditions. We used a CRISPR gene editing method[42] to knock down EED, following the procedures described in our previous study[43] with minor modifications. Briefly, the *Eed*-targeting single guide RNA (sgRNA) sequence (listed in Table S2) was cloned into lentiCRISPR v2 (Table S1). Lentivirus was produced in HEK293T cells, as we reported.[44] MOVAS cells were transduced with lentivirus for 3 days. We used mixed populations of cells for experiments to avoid a lengthy selection of single clones of permanent knockout that is prone to compensatory responses. We hence denoted the cell culture as EED knockdown (KD) rather than knockout.

SMC proliferation assay (CellTiter-Glo)

MOVAS cells were cultured in full medium (DMEM plus 10% FBS) and then starved in basal medium (0.5% FBS) overnight. The mitogen (human PDGF-BB, final 20 ng/mL) or solvent control (sterile 4 mM HCl and 0.1% BSA) was added. After 72 h of incubation, plates were decanted and refilled with 50 μL of CellTiter-Glo reagent/50 μL of PBS per well. The incubation continued for 10 min at room temperature, and the plates were then read in FlexStation 3 Benchtop Multi-Mode Microplate Reader (Molecular Devices, San Jose, CA) (250-ms integration). For pre-treatment, the inhibitor EED226 or JQ1 dissolved in DMSO or its vehicle control (equal volume of DMSO) mixed in fresh basal medium was added to the cell culture 4 h prior to mitogen (PDGF-BB) stimulation.

coIP

We followed the protocol that we used recently.[45] The plasmids of Lenti-FLAG-BRD4-GFP, Lenti-HA-EED, Lenti-FLAG-EED (and FLAG-tagged truncated EED constructs), Lenti-HA-STAT3-GFP, and Lenti-HA-EZH2-GFP were constructed and each was used to transfect HEK293 cells (Addgene, catalog no. 60360) in the presence of the JetPRIME transfection reagent. Cells were lysed on ice for 30 min in Pierce IP Lysis Buffer (Thermo Fisher Scientific, catalog no. 87788) containing Halt Protease Inhibitor Cocktail (87785; Thermo Fisher Scientific), and then centrifuged at 13,200 × g for 15 min at 4°C. The supernatant was incubated with 50 μL of Pierce Anti-DYKDDDDK Magnetic Agarose beads (A36797; Thermo Fisher Scientific) at 4°C overnight. The beads were washed 3× with cold PBS buffer and then incubated in 0.1 M glycine (pH 2.8) for 10 min at room temperature with frequent vortex to elute the immunoprecipitates. The eluate was neutralized with 1 M Tris-HCl, pH 8.5 (15 μL per 100 μL of eluate) and briefly heated at 95°C before its use for SDS-PAGE and western blot analysis.

ChIP followed by qPCR

We used the same protocol as in our recent report.[46] The Pierce Magnetic ChIP kit (26157; Thermo Fisher Scientific) was used. Briefly, protein-protein and protein-DNA interactions were stabilized via crosslink after adding formaldehyde (final concentration 1%) to the SMC culture. After a 10-min incubation, crosslinking was terminated with glycine. Cells were washed and lysed on ice. The nuclei were collected by centrifugation, digested with MNase at 37°C for 15 min for chromatin fragmentation, and MNase Stop Solution was then added. The nuclei were recovered by centrifugation at 9,000 × g for 5 min, re-suspended in IP Dilution Buffer, and then sonicated (four 5-s pulses at 20 W for 2 × 10^6 cells) to break the nuclear membrane. After another centrifugation at 9,000× g for 5 min, the supernatant was collected and incubated overnight with an antibody against endogenous EED or BRD4 (Table S3) or immunoglobulin (Ig) G control (5 μg of antibody per reaction). The EED or BRD4 ChIP mixture was then incubated with ChIP-grade Protein A/G Magnetic beads overnight at 4°C. The beads were collected and washed sequentially with IP Wash Buffer-1 and IP Wash Buffer-2, and the protein-DNA immunoprecipitates were eluted in the elution buffer. To reverse crosslink, 5 M NaCl was added, and RNAse A and Proteinase K were then used to digest RNA and protein. The DNA freed from the crosslink was purified with DNA Clean-Up Column and detected by qPCR. The primers (Table S4) were designed using the information from BRD4 ChIP sequencing.[20] Triplicate samples were used in each qPCR assay, which was performed using DNA samples from three independent ChIP experiments.

Western blot analysis

Samples were quantified for total protein contents using Pierce BCA Protein Assay Kit and loaded based on equal protein amount for SDS-PAGE. Separated proteins were electro-transferred to the polyvinylidene fluoride (PVDF) membrane for immunoblotting. The PVDF membrane was first incubated overnight at 4°C with a primary antibody (see the list in Table S3), and then with a peroxidase-conjugated secondary antibody (Table S3) for 1 h at room temperature. The protein bands of interest were detected using enhanced chemiluminescence reagents and recorded by Amersham, ImageQuant 800, GE company. Protein band densitometry was quantified using the ImageJ 64 software (https://imagej.nih.gov/ij/) and normalized to loading control and then to the basal (non-stimulated) condition in each experiment. The values from at least three independent repeat experiments were averaged to calculate mean ± SEM.

qRT-PCR

Following the manufacturer's instruction, we used the Trizol reagent to isolate total RNA from cell lysates, which was subjected to reverse transcription using the High-Capacity cDNA Reverse Transcription kit. The resulting cDNA was amplified by real-time quantitative PCR performed with Applied Biosystems Quant Studio 3 (Thermo Fisher Scientific) using Perfecta SYBR Green Master Fast Mix. Relative gene expression was determined by the delta-delta ct method using *Gapdh* as a housekeeping gene. qPCR was performed in triplicate. Primers are listed in Table S5.

Lentivector for EED gain of function in SMCs

To express EED in SMCs, a lentiviral vector was constructed for SMC transduction. Molecular cloning was performed using a GFP-expressing lentivector (empty vector [EV] control) to generate an EED-overexpression (OE) vector, following our recently published method.[44] Lentivirus was packaged in Lenti-x 293 cells as we described in detail.[44] Briefly, the crude viral solution was concentrated using Lenti-x concentrator (Takara, catalog no. 631232) to a final concentration of 108–109 IFU/mL using the Lenti-x qRT-PCR Titration Kit (Takara, catalog no. 631235). MOI was determined based on the fluorescence from GFP expression following the transduction with lentiviral vectors. MOI of 10 was optimal for SMC transduction without noticeable cytotoxic or cytostatic effects. Lentivirus and polybrene (Santa Cruz, catalog no. sc-134220) were added together into the SMC culture and incubated for 6 h followed by 24-h recovery in full medium. The infected SMCs were starved (0.5% FBS) for 6 h prior to stimulation with PDGF-BB.

Immunofluorescence staining on artery tissue sections

Fluorescent immunostaining was performed following our published protocol.[47] Briefly, artery sections were incubated with a primary antibody for 12 h and rinsed at least three times. The sections were then incubated with an anti-rabbit/mouse secondary antibody conjugated with Alexa Fluor 594 (Invitrogen, Carlsbad, CA, catalog no. A-11037/A-21203) and rinsed. The specific antigen was then visualized with fluorescence microscopy. Detailed information on antibodies is included in Table S3. For quantification, five immunostained sections from each animal were used. Fluorescence intensity in each image field was quantified by using ImageJ software and normalized to the number of DAPI-stained nuclei in the media and neointima layers. The values from all five sections were pooled to generate the mean for each animal. The means from all animals in each group were then averaged, and the final mean (±SEM) was calculated.

Statistical analysis

Data normality was assessed based on the Shapiro-Wilk normality test prior to statistical calculation. As specified in each figure legend, one-way analysis of variance (ANOVA) followed by *post hoc* test was applied to multi-group comparison; Student's t test was used for two-group comparisons. $p < 0.05$ was considered significant. The Prism 6.0 software (GraphPad) was used.

DATA AVAILABILITY

The data that support the findings of this study are available from the corresponding author upon reasonable request. Some data may not be made available because of privacy or ethical restrictions.

SUPPLEMENTAL INFORMATION

Supplemental information can be found online at https://doi.org/10.1016/j.omtn.2023.02.024.

ACKNOWLEDGMENTS

This work was supported by NIH R01 grants HL133665 (to L.-W.G.), HL143469 and HL129785 (to K.C.K. and L.-W.G.), and HL148239 and HL125912 to (C.L.M.). This work was also supported by an AHA post-doctoral fellowship award 20POST35210967 (to M.X.Z.) and a Leducq Foundation Transatlantic Network of Excellence Program grant, PlaqOmics (to C.L.M.).

AUTHOR CONTRIBUTIONS

M.Z. carried out most of the original experiments. J.L. performed the additional experiments required for manuscript revision. Q.W. contributed the ChIP-qPCR data. R.T. did immunofluorescence staining in the additional experiments. G.U. performed the animal surgery model. Y.H., J.V.M., and B.W. assisted with the *in vitro* and *in vivo* data collection. K.C.K., C.L.M., and L.-W.G. acquired funding and supervised research. L.-W.G. wrote the manuscript. L.-W.G. and C.L.M. critically reviewed and revised the manuscript.

DECLARATION OF INTERESTS

The authors declare no competing interests.

REFERENCES

1. Shi, N., Mei, X., and Chen, S.Y. (2019). Smooth muscle cells in vascular remodeling. Arterioscler. Thromb. Vasc. Biol. *39*, e247–e252.
2. Zain, M.A., Jamil, R.T., and Siddiqui, W.J. (2021). Neointimal Hyperplasia. In StatPearls (StatPearls Publishing).
3. Libby, P. (2021). The changing landscape of atherosclerosis. Nature *592*, 524–533.
4. Zaware, N., and Zhou, M.M. (2017). Chemical modulators for epigenome reader domains as emerging epigenetic therapies for cancer and inflammation. Curr. Opin. Chem. Biol. *39*, 116–125.
5. Borck, P.C., Guo, L.W., and Plutzky, J. (2020). BET epigenetic reader proteins in cardiovascular transcriptional programs. Circ. Res. *126*, 1190–1208.

6. Bhat, K.P., Ümit Kaniskan, H., Jin, J., and Gozani, O. (2021). Epigenetics and beyond: targeting writers of protein lysine methylation to treat disease. Nat. Rev. Drug Discov. *20*, 265–286.

7. Kouznetsova, V.L., Tchekanov, A., Li, X., Yan, X., and Tsigelny, I.F. (2019). Polycomb repressive 2 complex-Molecular mechanisms of function. Protein Sci. *28*, 1387–1399.

8. Piunti, A., and Shilatifard, A. (2021). The roles of Polycomb repressive complexes in mammalian development and cancer. Nat. Rev. Mol. Cell Biol. *22*, 326–345.

9. Bradner, J.E., Hnisz, D., and Young, R.A. (2017). Transcriptional addiction in cancer. Cell *168*, 629–643.

10. Tang, P., Zhang, J., Liu, J., Chiang, C.M., and Ouyang, L. (2021). Targeting bromodomain and extraterminal proteins for drug discovery: from current progress to technological development. J. Med. Chem. *64*, 2419–2435.

11. Wang, B., Zhang, M., Takayama, T., Shi, X., Roenneburg, D.A., Kent, K.C., and Guo, L.W. (2015). BET bromodomain blockade mitigates intimal hyperplasia in rat carotid arteries. EBioMedicine *2*, 1650–1661.

12. Dutzmann, J., Haertlé, M., Daniel, J.M., Kloss, F., Musmann, R.J., Kalies, K., Knöpp, K., Pilowski, C., Sirisko, M., Sieweke, J.T., et al. (2021). BET bromodomain containing epigenetic reader proteins regulate vascular smooth muscle cell proliferation and neointima formation. Cardiovasc. Res. *117*, 850–862.

13. Yang, C.Y., and Wang, S. (2017). Allosteric inactivation of polycomb repressive complex 2 (PRC2) by inhibiting its adapter protein: embryonic ectodomain development (EED). J. Med. Chem. *60*, 2212–2214.

14. Margueron, R., Justin, N., Ohno, K., Sharpe, M.L., Son, J., Drury, W.J., 3rd, Voigt, P., Martin, S.R., Taylor, W.R., De Marco, V., et al. (2009). Role of the polycomb protein EED in the propagation of repressive histone marks. Nature *461*, 762–767.

15. Martin, M.C., Zeng, G., Yu, J., and Schiltz, G.E. (2020). Small molecule approaches for targeting the polycomb repressive complex 2 (PRC2) in cancer. J. Med. Chem. *63*, 15344–15370.

16. Qi, W., Zhao, K., Gu, J., Huang, Y., Wang, Y., Zhang, H., Zhang, M., Zhang, J., Yu, Z., Li, L., et al. (2017). An allosteric PRC2 inhibitor targeting the H3K27me3 binding pocket of EED. Nat. Chem. Biol. *13*, 381–388.

17. Liang, J., Li, Q., Cai, W., Zhang, X., Yang, B., Li, X., Jiang, S., Tian, S., Zhang, K., Song, H., et al. (2019). Inhibition of polycomb repressor complex 2 ameliorates neointimal hyperplasia by suppressing trimethylation of H3K27 in vascular smooth muscle cells. Br. J. Pharmacol. *176*, 3206–3219.

18. Lino Cardenas, C.L., Kessinger, C.W., Chou, E., Ghoshhajra, B., Yeri, A.S., Das, S., Weintraub, N.L., Malhotra, R., Jaffer, F.A., and Lindsay, M.E. (2019). HDAC9 complex inhibition improves smooth muscle-dependent stenotic vascular disease. JCI Insight *4*, e124706.

19. Zhang, M.W.B., Urabe, G., Shi, X., and Guo, L.-W. (2017/5). Inhibition of the enhancer of zeste homolog family mitigates intimal hyperplasia in rat carotid arteries. Arterioscler. Thromb. Vasc. Biol. *37*, A332.

20. Zhang, M.W.B., Urabe, G., Ozer, H.G., Han, R., Kent, K.C., and Guo, L.-W. (2020). Angioplasty-induced epigenomic remodeling entails BRD4 and EZH2 hierarchical regulations. Preprint at bioRxiv. https://doi.org/10.1101/2020.03.12.989640.

21. Clowes, A.W., Reidy, M.A., and Clowes, M.M. (1983). Mechanisms of stenosis after arterial injury. Lab. Invest. *49*, 208–215.

22. Marx, S.O., Totary-Jain, H., and Marks, A.R. (2011). Vascular smooth muscle cell proliferation in restenosis. Circ. Cardiovasc. Interv. *4*, 104–111.

23. He, Y., Selvaraju, S., Curtin, M.L., Jakob, C.G., Zhu, H., Comess, K.M., Shaw, B., The, J., Lima-Fernandes, E., Szewczyk, M.M., et al. (2017). The EED protein-protein interaction inhibitor A-395 inactivates the PRC2 complex. Nat. Chem. Biol. *13*, 389–395.

24. Devaiah, B.N., Case-Borden, C., Gegonne, A., Hsu, C.H., Chen, Q., Meerzaman, D., Dey, A., Ozato, K., and Singer, D.S. (2016). BRD4 is a histone acetyltransferase that evicts nucleosomes from chromatin. Nat. Struct. Mol. Biol. *23*, 540–548.

25. Devaiah, B.N., Gegonne, A., and Singer, D.S. (2016). Bromodomain 4: a cellular Swiss army knife. J. Leukoc. Biol. *100*, 679–686.

26. Han, Z., Xing, X., Hu, M., Zhang, Y., Liu, P., and Chai, J. (2007). Structural basis of EZH2 recognition by EED. Structure *15*, 1306–1315.

27. Qin, A.C., Jin, H., Song, Y., Gao, Y., Chen, Y.F., Zhou, L.N., Wang, S.S., and Lu, X.S. (2020). The therapeutic effect of the BRD4-degrading PROTAC A1874 in human colon cancer cells. Cell Death Dis. *11*, 805.

28. Filippakopoulos, P., Qi, J., Picaud, S., Shen, Y., Smith, W.B., Fedorov, O., Morse, E.M., Keates, T., Hickman, T.T., Felletar, I., et al. (2010). Selective inhibition of BET bromodomains. Nature *468*, 1067–1073.

29. Zhang, M., Urabe, G., Ozer, H.G., Xie, X., Webb, A., Shirasu, T., Li, J., Han, R., Kent, K.C., Wang, B., and Guo, L.W. (2022). Angioplasty induces epigenomic remodeling in injured arteries. Life Sci. Alliance *5*, e202101114.

30. Wang, Y.C., Cui, X.B., Chuang, Y.H., and Chen, S.Y. (2017). Janus kinase 3, a novel regulator for smooth muscle proliferation and vascular remodeling. Arterioscler. Thromb. Vasc. Biol. *37*, 1352–1360.

31. Daniel, J.M., Dutzmann, J., Bielenberg, W., Widmer-Teske, R., Gündüz, D., Hamm, C.W., and Sedding, D.G. (2012). Inhibition of STAT3 signaling prevents vascular smooth muscle cell proliferation and neointima formation. Basic Res. Cardiol. *107*, 261.

32. Wang, Q., Jia, S., Wang, D., Chen, X., Kalvakolanu, D.V., Zheng, H., Wei, X., Wen, N., Liang, H., Guo, B., and Zhang, L. (2020). A combination of BRD4 and HDAC3 inhibitors synergistically suppresses glioma stem cell growth by blocking GLI1/IL6/STAT3 signaling Axis. Mol. Cancer Ther. *19*, 2542–2553.

33. Park, H.S., Quan, K.T., Han, J.H., Jung, S.H., Lee, D.H., Jo, E., Lim, T.W., Heo, K.S., Na, M., and Myung, C.S. (2017). Rubiarbonone C inhibits platelet-derived growth factor-induced proliferation and migration of vascular smooth muscle cells through the focal adhesion kinase, MAPK and STAT3 Tyr(705) signalling pathways. Br. J. Pharmacol. *174*, 4140–4154.

34. Guo, Z.Y., Hao, X.H., Tan, F.F., Pei, X., Shang, L.M., Jiang, X.L., and Yang, F. (2011). The elements of human cyclin D1 promoter and regulation involved. Clin. Epigenetics *2*, 63–76.

35. Rossi, M.N., Andresini, O., Matteini, F., and Maione, R. (2018). Transcriptional regulation of p57kip2 expression during development, differentiation and disease. Front. Biosci. *23*, 83–108.

36. Felisbino, M.B., and McKinsey, T.A. (2018). Epigenetics in cardiac fibrosis: emphasis on inflammation and fibroblast activation. JACC. Basic Transl. Sci. *3*, 704–715.

37. Pelham-Webb, B., Murphy, D., and Apostolou, E. (2020). Dynamic 3D chromatin reorganization during establishment and maintenance of pluripotency. Stem Cell Rep. *15*, 1176–1195.

38. Huang, X., Yan, J., Zhang, M., Wang, Y., Chen, Y., Fu, X., Wei, R., Zheng, X.L., Liu, Z., Zhang, X., et al. (2018). Targeting epigenetic crosstalk as a therapeutic strategy for EZH2-aberrant solid tumors. Cell *175*, 186–199.e19.

39. Crump, N.T., Ballabio, E., Godfrey, L., Thorne, R., Repapi, E., Kerry, J., Tapia, M., Hua, P., Lagerholm, C., Filippakopoulos, P., et al. (2021). BET inhibition disrupts transcription but retains enhancer-promoter contact. Nat. Commun. *12*, 223.

40. Li, X., Gera, L., Zhang, S., Chen, Y., Lou, L., Wilson, L.M., Xie, Z.R., Sautto, G., Liu, D., Danaher, A., et al. (2021). Pharmacological inhibition of noncanonical EED-EZH2 signaling overcomes chemoresistance in prostate cancer. Theranostics *11*, 6873–6890.

41. Neele, A.E., Willemsen, L., Chen, H.J., Dzobo, K.E., and de Winther, M.P.J. (2020). Targeting epigenetics as atherosclerosis treatment: an updated view. Curr. Opin. Lipidol. *31*, 324–330.

42. Sanjana, N.E., Shalem, O., and Zhang, F. (2014). Improved vectors and genome-wide libraries for CRISPR screening. Nat. Methods *11*, 783–784.

43. Yang, H., Shen, H., Li, J., and Guo, L.W. (2019). SIGMAR1/Sigma-1 receptor ablation impairs autophagosome clearance. Autophagy *15*, 1539–1557.

44. Zhang, M., Wang, B., Urabe, G., Huang, Y., Plutzky, J., Kent, K.C., and Guo, L.W. (2019). The BD2 domain of BRD4 is a determinant in EndoMT and vein graft neointima formation. Cell. Signal. *61*, 20–29.

45. Zhang, M., Urabe, G., Little, C., Wang, B., Kent, A.M., Huang, Y., Kent, K.C., and Guo, L.W. (2018). HDAC6 regulates the MRTF-A/SRF Axis and vascular smooth muscle cell plasticity. JACC. Basic Transl. Sci. *3*, 782–795.

46. Wang, Q., Ozer, H.G., Wang, B., Zhang, M., Urabe, G., Huang, Y., Kent, K.C., and Guo, L.W. (2021). A hierarchical and collaborative BRD4/CEBPD partnership governs vascular smooth muscle cell inflammation. Mol. Ther. Methods Clin. Dev. *21*, 54–66.

47. Shi, X., Guo, L.W., Seedial, S., Takayama, T., Wang, B., Zhang, M., Franco, S.R., Si, Y., Chaudhary, M.A., Liu, B., and Kent, K.C. (2016). Local CXCR4 upregulation in the injured arterial wall contributes to intimal hyperplasia. Stem Cells *34*, 2744–2757.

OMTN, Volume *31*

Supplemental information

Gene-repressing epigenetic reader EED unexpectedly enhances cyclinD1 gene activation

Mengxue Zhang, Jing Li, Qingwei Wang, Go Urabe, Runze Tang, Yitao Huang, Jose Verdezoto Mosquera, K. Craig Kent, Bowen Wang, Clint L. Miller, and Lian-Wang Guo

Supplemental Figures

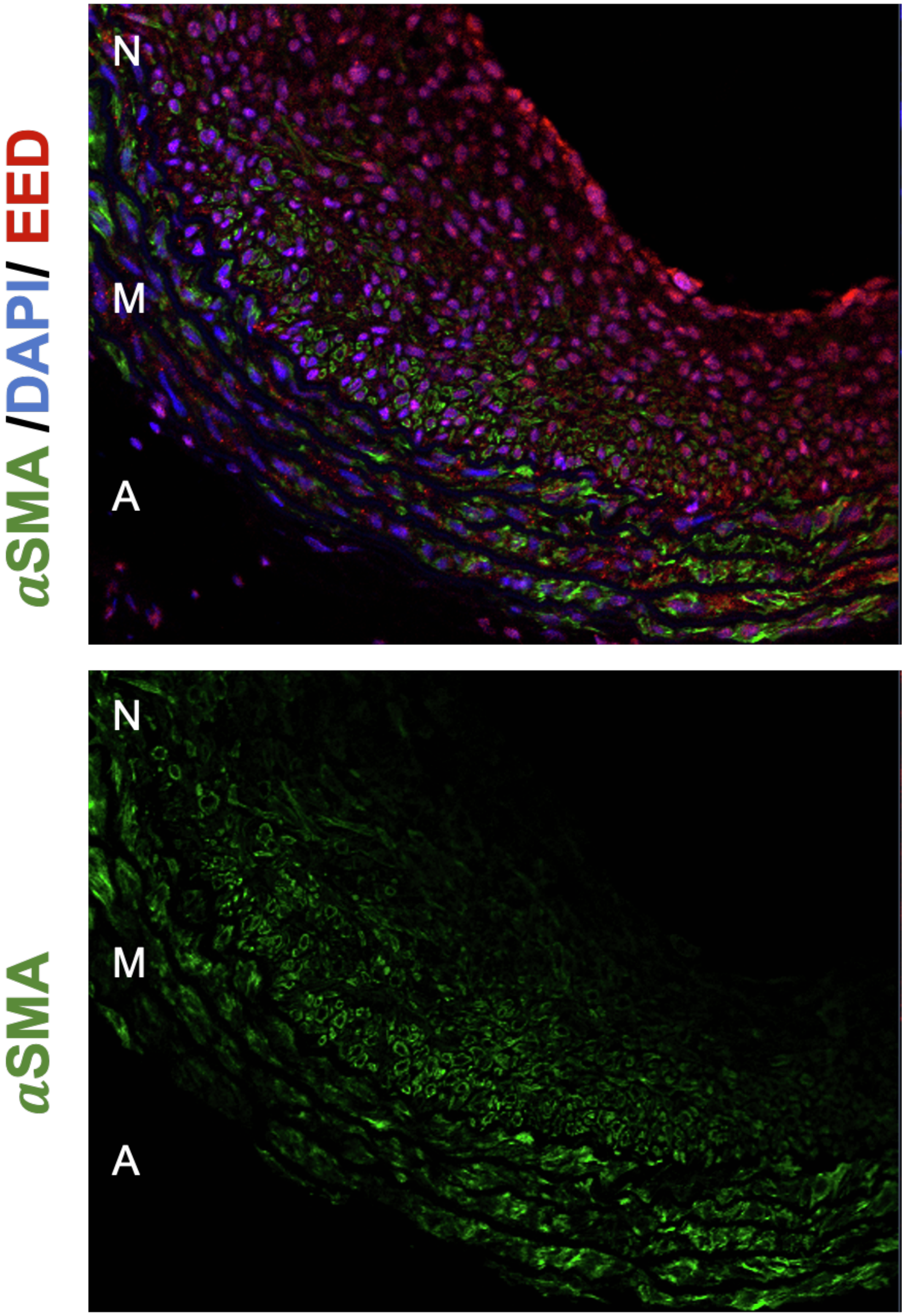

Figure S1. Enlarged view of EED and αSMA co-staining.
Please refer to the Figure 1C confocal images. The picture of merged colors shows EED in the nucleus surrounded by αSMA in the cytosol.

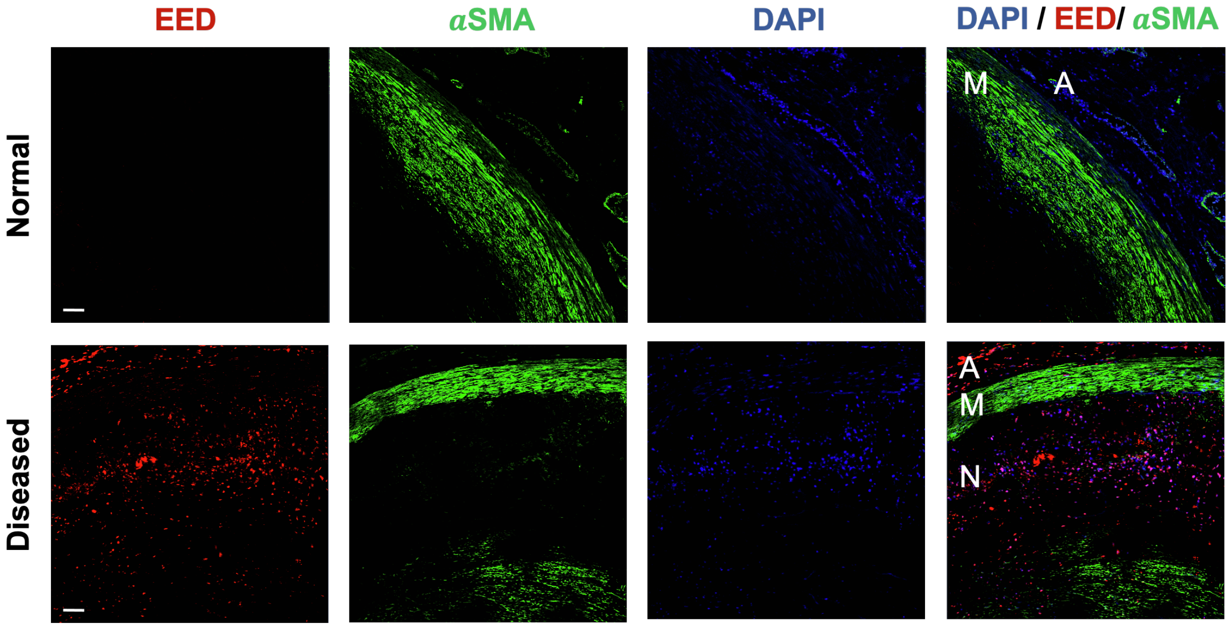

Figure S2. Co-staining of EED and αSMA.
Please refer to the Figure 2D confocal images. Shown here are all the images of individual color channels.

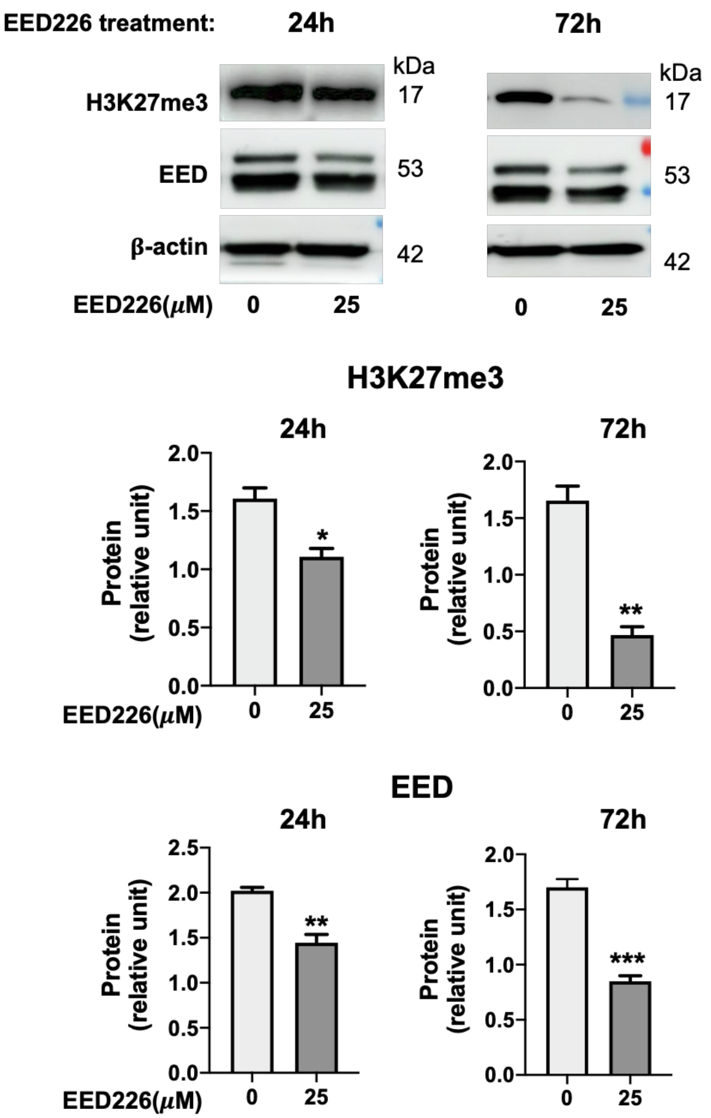

Figure S3. EED-selective inhibitor EED226 reduces SMC H3K27me3 levels
Starved MOVAS mouse SMCs were incubated with EED226 (25 μM) or equal amount of DMSO (0 μM) for indicated hours before harvest for immunoblot analysis. Student's t-test: *$P<0.05$, **$P<0.01$, ***$P<0.001$.

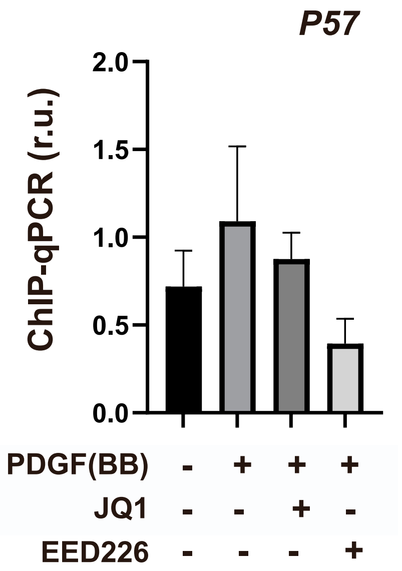

Figure S4. ChIP-qPCR to detect the distal side of the *P57* promoter

MOVAS cells were cultured, starved, preincubated with EED226 or JQ1 (1 μM), and then stimulated with PDGF-BB. Cells were harvested for assays after 48h PDGF stimulation. ChIP was performed using an antibody specific for endogenous EED or endogenous BRD4, as described for Figure 6. qPCR was performed using primers for a region on the distal side of the *P57* promoter (~ -2034bp-1885bp from TSS). Quantification: Three independent ChIP experiments were performed in separate times (n = 3). The qPCR reading was first normalized to *Gapdh* using the delta-delta ct method, and then to the basal-condition (no PDGF-BB, no inhibitor) value from one of the 3 repeat experiments. All readings were thereby converted to fold changes and averaged to produce mean ± SEM. Statistics: One-way ANOVA and Bonferroni post-hoc test. No significance was found ($P > 0.05$).

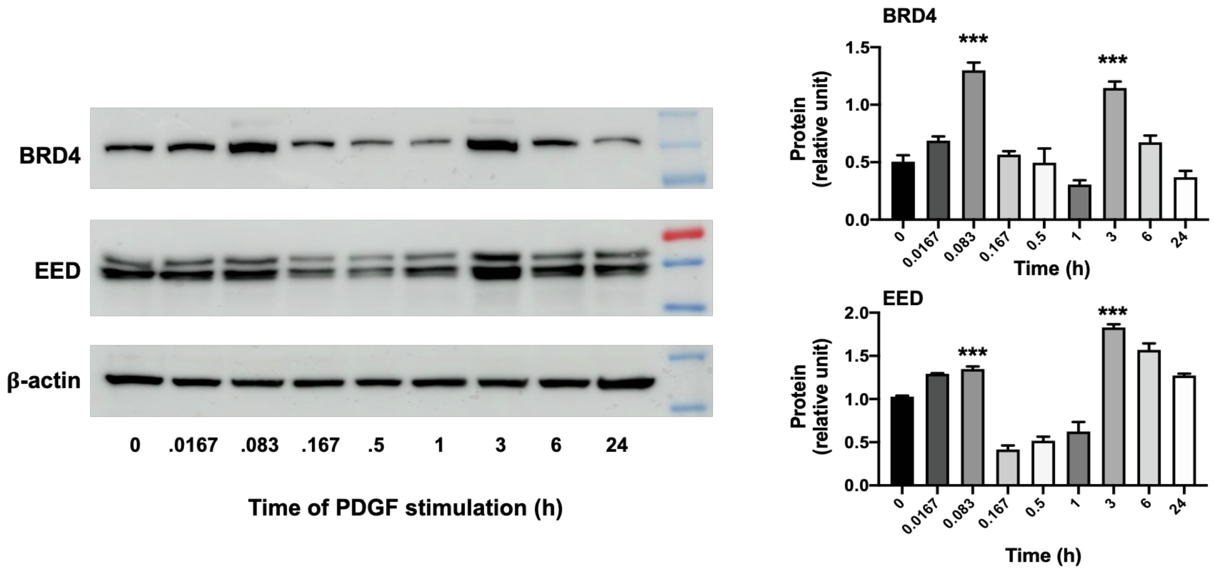

Figure S5. PDGF-induced EED and BRD4 protein level changes in SMCs

Starved MOVAS mouse SMCs were stimulated with 20 ng/ml PDGF-BB for indicated hours before immunoblotting assay. Densitometry was normalized to loading control (β-actin). The data values from 3 independent repeat experiments were averaged to calculate mean ± SEM (n = 3). Statistics: One-way analysis of variance (ANOVA) followed by Bonferroni post-hoc test; ***P<0.001, compared to the basal condition without PDGF-BB (the first bar in each plot).

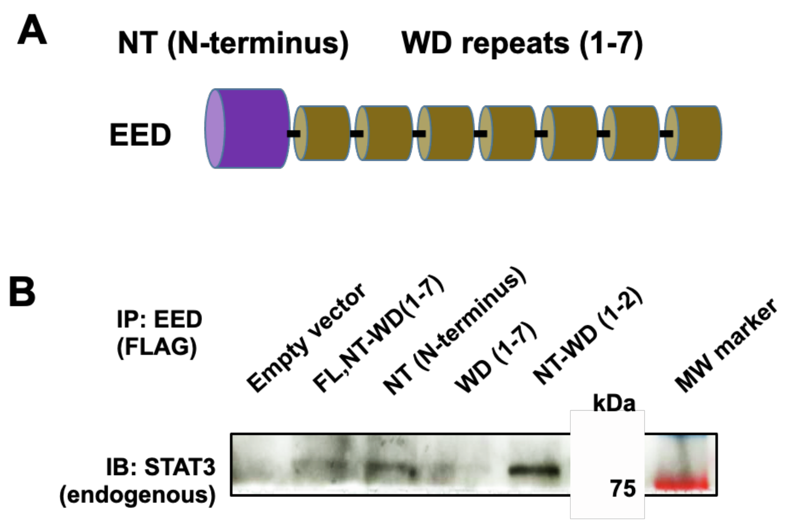

Figure S6. The **EED N-terminus is mainly responsible for its interaction with STAT3**

A. Diagram of the domains of the EED protein.
B. B. Co-IP. FLAG-tagged EED constructs were expressed in HEK293A cells, and an anti-FLAG antibody was used for IP. An antibody for endogenous STAT3 was used for immunoblotting (IB). FL, full length, including the N-terminus (NT) and 7 WD repeats.

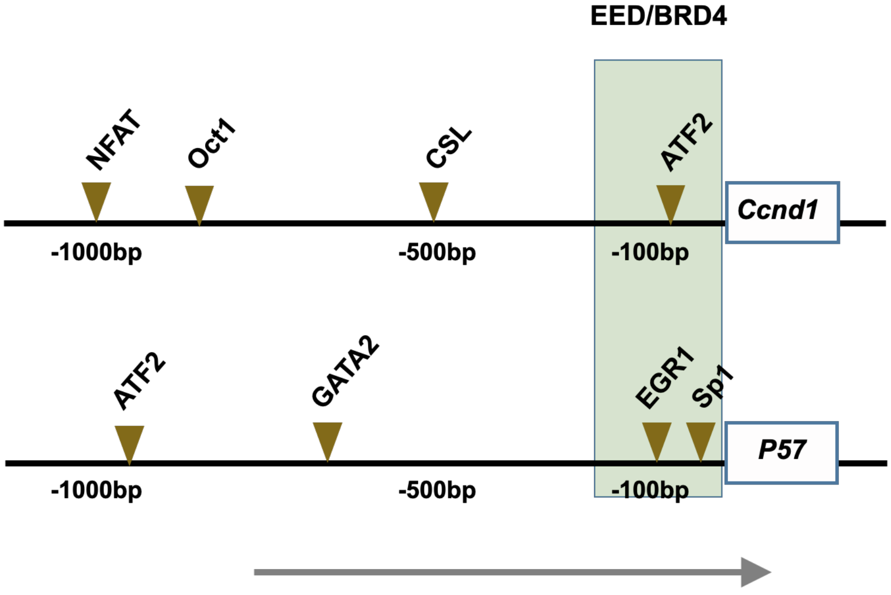

Figure S7. Transcription factors that possibly bind *Ccnd1* and *P57* promoters

The box marks the promoter regions where EED and BRD4 are enriched.

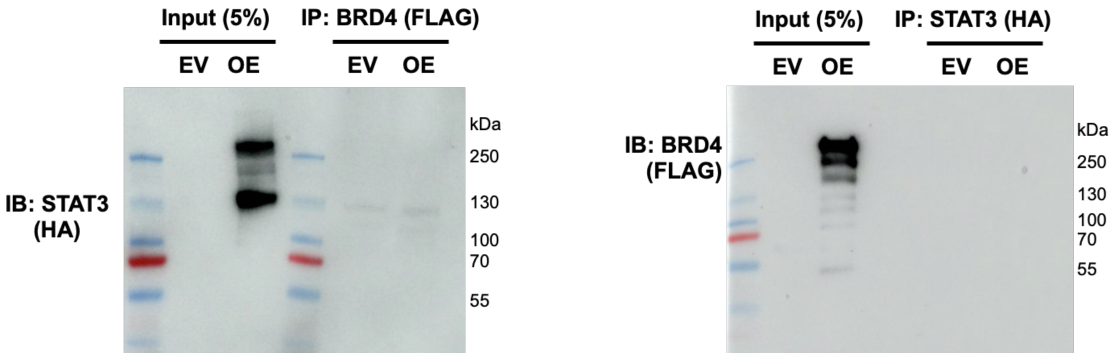

Figure S8. Lack of BRD4/EZH2 co-immunoprecipitation

The assay was performed as described for Figure 3. Briefly, HEK293 cells were transfected with plasmids detailed in Methods. An anti-HA or anti-FLAG antibody was used for IP or the corresponding IB. EV: empty vector.

Supplemental Tables

Table S1. Major resources

Product	Manufacturer	Catalog Number
MOVAS cells	ATCC	CRL-2797
DMEM	Thermo Fisher Scientific	10313039
PDGF-BB	R&D	520-BB
Lipofectamine RNAiMAX Transfection Reagent	Invitrogen	3778150
JetPRIME Transfection reagent	Polyplus transfection	114-07
PageRuler™ PLUS Prestained Protein Ladder	Thermo Fisher Scientific	26620
Clarity Western ECL Substrate	Bio-Rad	170-5060
QIAGEN Plasmid MIDIprep Kit	QIAGEN	12143
PureLink™ Quick Plasmid Miniprep Kit	Invitrogen	K210011
PureLink™ Quick Gel Extraction and PCR Purification Combo Kit	Invitrogen	K220001
lentiCRISPR v2	addgene	52961
Lenti-x qRT-PCR Titration Kit	Takara	631235
Trizol Reagent	Thermo Fisher Scientific	15596026
Perfecta SYBR Green Master Fast Mix	VWR	101414-286

Pierce™ Protein A/G Magnetic Agarose Beads	Invitrogen	78610
UltraPure™ Salmon Sperm DNA Solution	Invitrogen	15632011
CellTiter-Glo kit	Promega	G7570
Anti-HA Magnetic Agarose beads	Invitrogen	88836
Pierce™ Anti-DYKDDDDK (FLAG) Magnetic Agarose	Thermo Fisher Scientific	A36797
Mouse IgG	Invitrogen	10400C
High-Capacity cDNA Reverse Transcription kit	Thermo Fisher Scientific	4368814
Pierce BCA Protein Assay kit,	Thermo Fisher Scientific,	23227
EED226 (in vitro use)	Selleckchem	S8496
EED226 (in vivo use)	Chemietek	CT-EED226
A-395	Sigma-Aldrich	SML1923
JQ1	Apexbio	A1910

Table S2. sgRNAs and siRNAs for knockdown

Name	Sequence (5’-3’)
EED sgRNA	Sense: GGAAGTGTCGACTGCGCCGG
	Antisense: CCGGCGCAGTCGACACTTCC
STAT3 shRNA#1	Target Sequence: CCAACATCTGCCTGGACCGTCTG
STAT3 shRNA#2	Target Sequence: TCAGGTTGCTGGTCAAATTTC
STAT3 shRNA#3	Target Sequence: ACATGGAGGAGTCTAACAA

Table S3. Antibodies for Western blot (WB), ChIP, IP, or immunofluorescence (IF)

Antigen	Manufacturer	Catalog Number	Dilution Ratio	Application
Beta-actin	Proteintech	60008-1-Ig	1:10000	WB
FLAG tag	Sigma-Aldrich	F3165	1:1000	WB/IP
HA tag	Cell Signaling Technology	3724	1:1000	WB/IP
Histone-3	Cell Signaling Technology	14269	1:1000	WB
H3K27me3	Cell Signaling Technology	4909	1:1000	WB
H3K27ac	Active Motif	39133	1:1000	WB
BRD4	Abcam	Ab128874	1:1000	WB
BRD4	Abcam	Ab128874	1:1000	ChIP
EED	R&D	AF5827	1:500	WB
EED	Cell Signaling Technology	85322S	1:1000	ChIP
EED	LSBio	LS-C153641	1:100	IF
EZH2	Cell Signaling Technology	5246	1:1000	WB
STAT3	Cell Signaling Technology	9139	1:1000	WB
P-STAT3	Cell Signaling Technology	9145	1:1000	WB
Cyclin-D1	Cell Signaling Technology	55506	1:1000	WB
Cyclin-D1	Cell Signaling Technology	55506	1:100	IF
p-STAT3	Novus	NB100-82213	1:100	IF

Table S4. Primers for ChIP-qPCR

Mouse *Ccnd1* (Region: -176 to -18 bp)	Forward: GGGGAGTTTTGTTGAAGTTGC
	Reverse: GGGCTGTGGTCTCGGTTG
Mouse *P57* (Region: ~ -183 to -2 bp)	Forward: AGTGCGCTGTGCTCGAGG
	Reverse: GTCGAAGGCTGTGCAAACG
Mouse *P57* (Region: ~ -2034 to -1885 bp)	Forward: TGCTTCTCCCAACAAATAG
	Reverse: CTCCCCAACAGTCCAAAT
Mouse *Gapdh*	Forward: AGGTCGGTGTGAACGGATTTG
	Reverse: TGTAGACCATGTAGTTGAGGTCA

Table S5. Primers for qRT-PCR

Ccnd1(CyclinD1)	Forward: AGAGGCGGATGAGAACAAGC
	Reverse: CCTTGTTTAGCCAGAGGCCG
Cdkn1c (P57)	Forward: GCCAATGCGAACGACTTCTT
	Reverse: GTTTGGAGAGGGACACCCTG
Gapdh	Forward: GAGAGTGTTTCCTCGTCCCG
	Reverse: ATGGGCTTCCCGTTGATGAC
Stat3	Forward: GGGCCTGGTGTGAACTACTC
	Reverse: ACATCGGCAGGTCAATGGTA
